# Effects of Different Concentrations of Platelet-Rich Plasma-Derived Extracellular Vesicles on Cellular Activity and Lineage-Associated Marker Expression in Tendon Stem Cells

**DOI:** 10.3390/cells15212008

**Published:** 2026-11-04

**Authors:** Simao Xu, Jingzhi Zhang, Benxiang He

**Affiliations:** 1 College of Physical Education and Health, Guangxi Normal University, Guilin 541000, China; xusimao666@163.com; 2 Institute of Sports Science, Guangxi Normal University, Guilin 541000, China; 3 Sichuan Academy of Chinese Medicine Sciences, Chengdu 610000, China

**Keywords:** platelet-rich plasma, extracellular vesicles, tendon stem cells, tendon-associated markers, lineage-associated markers

## Abstract

Background: Tendon stem cells (TSCs) contribute to tendon maintenance and repair through their self-renewal capacity and multilineage differentiation potential. Platelet-rich plasma-derived extracellular vesicles (PRP-EVs) may regulate tissue repair; however, their effects on TSC cellular activity and lineage-associated marker expression remain unclear. Objective: To investigate the effects of different concentrations of PRP-EVs on CCK-8 signal and the expression of tendon-associated and alternative lineage-associated markers in TSCs. Methods: Rat TSCs were isolated from Achilles tendon tissue by enzymatic digestion and characterized using flow cytometry, immunofluorescence staining, colony formation assays, and multilineage differentiation assays. PRP-EVs were isolated from pooled rat PRP and characterized using transmission electron microscopy (TEM), dynamic light scattering (DLS), and Western blotting. TSCs were treated with PRP-EVs at concentrations of 0, 5, 20, and 40 μg/mL. CCK-8 absorbance was measured as an indicator of cellular metabolic activity. The mRNA and protein expression levels of tendon-associated markers (ScxA, Col I, and Tnmd) and alternative lineage-associated markers (Osx, RUNX2, SOX9, and PPARγ) were assessed using quantitative real-time PCR and Western blotting, respectively. Results: TEM revealed vesicle-like structures in the PRP-EV preparation, with a mean particle diameter of 198.6 ± 94.5 nm measured by DLS. Western blotting detected the EV-associated proteins TSG101, CD9, and CD81. PRP-EV treatment increased the CCK-8 signal, with 40 μg/mL generally producing the strongest overall response among the concentrations tested, although differences between concentrations were not consistently significant. Treatment was also associated with increased expression of tendon-associated markers and generally decreased expression of alternative lineage-associated markers. Conclusion: PRP-EV treatment increased the CCK-8 signal and altered the expression of tendon-associated and alternative lineage-associated markers in cultured rat TSCs. These findings indicate that PRP-EVs modulate the molecular phenotype of TSCs in vitro.

## 1. Introduction

Tendon injuries, particularly chronic tendinopathies, are common musculoskeletal disorders that can substantially impair physical function, mobility, and quality of life [1]. Tendons are specialized connective tissues that transmit mechanical forces from muscle to bone and are essential for efficient movement [2]. However, their relatively low cellularity and limited vascularity contribute to their restricted intrinsic healing capacity, particularly after severe or chronic injury [3]. Tendon healing often involves prolonged tissue remodeling, disorganized extracellular matrix deposition, and the formation of mechanically inferior scar tissue, which may compromise the restoration of normal tendon structure and function [4]. Current management strategies, including pharmacological treatment, physical therapy, and exercise-based rehabilitation, can alleviate symptoms and improve function in some patients, but satisfactory structural and functional recovery remains challenging, especially in chronic tendinopathy [5,6]. These limitations have prompted increasing interest in biological approaches that may improve the quality of tendon repair and support tissue regeneration [1].

Tendon stem cells (TSCs), also referred to as tendon stem/progenitor cells (TSPCs), are a resident stem/progenitor cell population within tendon tissue and have attracted interest for their potential role in tendon maintenance and repair [7]. TSCs exhibit stem cell-associated properties, including clonogenicity and multilineage differentiation potential, and express both stem cell-associated and tendon-associated markers [8]. Their capacity for self-renewal and acquisition of a tendon-associated phenotype makes them relevant to the endogenous repair of injured tendons [9]. However, under pathological or inappropriate microenvironmental conditions, TSCs may also exhibit osteogenic, chondrogenic, or adipogenic differentiation, potentially contributing to aberrant tissue remodeling and impairing tendon homeostasis [10]. Therefore, identifying strategies that support TSC activity while favoring a tendon-associated molecular profile and limiting alternative lineage-associated phenotypes may be relevant to improving tendon repair.

Platelet-rich plasma (PRP) has attracted considerable attention as a biological treatment for tendon injury because it contains a high concentration of platelets and a complex mixture of bioactive components involved in tissue repair. Upon platelet activation, platelets release growth factors and other signaling molecules that can influence cell proliferation, collagen synthesis, angiogenesis, and extracellular matrix remodeling, providing a biological rationale for investigating PRP in tendon repair [11]. Previous studies have shown that PRP can enhance TSC proliferation and migration and increase the expression of tendon-associated genes and proteins, suggesting that PRP can modulate TSC behavior [12]. PRP has also been investigated for various tendinopathies, although clinical outcomes have varied across tendon sites and treatment protocols [13]. This heterogeneity is associated with differences in PRP preparation, platelet and leukocyte composition, dosage, and delivery methods, as well as the complex mixture of bioactive components within PRP. Identifying the components that contribute to PRP-mediated effects on TSCs may therefore help clarify its biological activity and provide a more defined basis for developing tendon repair strategies.

Extracellular vesicles (EVs) are membrane-bound particles that mediate intercellular communication through the transfer of bioactive proteins, lipids, and nucleic acids to recipient cells [14]. PRP-derived EV (PRP-EV) preparations have attracted increasing interest as potential cell-free approaches to tissue repair [15]. Preclinical studies have reported beneficial effects of PRP-derived EV preparations on tendon-bone healing and tendon repair [16]. PRP-EV preparations have also been shown to mitigate stress-associated TSPC senescence, preserve proliferation and tendon-associated marker expression, and improve tendon-bone healing in vivo [17]. More recently, PRP-EV preparations were reported to enhance TSPC proliferation, migration, and tendon-associated differentiation under inflammatory conditions and to improve histological and biomechanical outcomes in a rat model of insertional Achilles tendinopathy [18]. Despite these findings, existing studies have largely focused on proliferation, migration, or tenogenic differentiation, whereas the effects of PRP-exos on the broader differentiation profile of TSCs remain less clearly defined. In particular, whether PRP-exos can simultaneously favor a tendon-associated phenotype while suppressing osteogenic, chondrogenic, and adipogenic lineage-associated phenotypes, and whether these effects vary across different concentrations of PRP-exos, have not been systematically evaluated. Defining these effects may provide a more comprehensive view of how PRP-exos regulate TSC biological behavior and may further clarify their potential in tendon regeneration.

Therefore, this study investigated the effects of different concentrations of PRP-EVs on the biological behavior of rat TSCs in vitro. The CCK-8 assay was used to assess changes in the CCK-8 signal, while changes in tendon-associated and alternative lineage-associated marker expression were evaluated at both the mRNA and protein levels. Specifically, we examined ScxA, Tnmd, and Col I as tendon-associated markers, together with Osx and RUNX2 as osteogenic markers, SOX9 as a chondrogenic marker, and PPARγ as an adipogenic marker. We hypothesized that PRP-EV treatment would increase the CCK-8 signal, favor a tendon-associated molecular expression profile, and reduce the expression of alternative lineage-associated markers, with more pronounced effects at higher concentrations within the tested range.

## 2. Materials and Methods

### 2.1. Experimental Animals

Thirteen healthy male Sprague–Dawley rats (6 weeks old, specific pathogen-free grade; 179–198 g) were obtained from Jinan Pengyue Experimental Animal Breeding Co., Ltd. (License No. SCXK [Lu] 2022-0006, Jinan, China). Upon arrival, the rats were allowed to acclimatize for 1 week under standard housing conditions, with free access to food and water, a 12-h light/dark cycle, adequate ventilation, and a room temperature of 23–26 °C. After acclimatization, three rats were used for TSC isolation. TSCs derived from these three rats were combined to generate a single pooled TSC preparation, which was subsequently used for all four treatment groups. The remaining 10 rats served as PRP donors for PRP-EV preparation. All animal procedures, including Achilles tendon tissue collection for TSC isolation and blood collection by right ventricular puncture under anesthesia for PRP-EV preparation, were approved by the Experimental Animal Ethics Committee of Chengdu Sport University (Approval No. CDSU Ethics [2022] 79).

### 2.2. Isolation, Culture, and Preparation of TSCs

Rat TSCs were isolated from Achilles tendons by enzymatic digestion and cultured in vitro. Following anesthesia with 5% chloral hydrate (350 mg/kg), the hind limbs were aseptically collected and disinfected with 75% ethanol for 5 min. After rinsing with phosphate-buffered saline (PBS), the Achilles tendons were carefully dissected, and the tendon sheaths and surrounding paratendons were removed. The tendon tissues were minced and digested with type I collagenase (3 mg/mL) and neutral protease (4 mg/mL) at 37 °C for 1.5 h. The digested suspension was filtered through a 200-μm cell strainer, and the cells were collected by centrifugation at 1500 rpm for 6 min. Cells obtained from the three rat donors were pooled to generate a single TSC preparation, which was subsequently expanded and used across all four treatment groups.

The cell pellet was resuspended in α-MEM (Meilunbio, Shanghai, China; Cat. No. PWL007) supplemented with 15% fetal bovine serum (FBS, Gibco, Waltham, MA, USA) and 1% penicillin/streptomycin (Gibco, Waltham, USA) and cultured in T25 flasks at 37 °C in a humidified atmosphere containing 5% CO_2_. The medium was partially changed on day 3 and subsequently every 3 days. Cell morphology and growth were monitored using an inverted microscope (DSZ2000X inverted white-light microscope, UOP, Chongqing, China). During expansion, cells were passaged when the predefined cell fusion rate reached 80%. Cells directly isolated from tendon tissue were designated as P0, followed by P1, P2, and P3 after successive passages.

When P3 TSCs reached >80% confluence, the cells were washed once with PBS and detached with 1 mL trypsin-EDTA. Digestion was terminated when the cells retracted and rounded, with some cells beginning to detach. Complete medium was then added to obtain a single-cell suspension. An aliquot was used for TSC characterization, while the remaining cells were collected by centrifugation at 1500 rpm for 5 min and resuspended in cryopreservation medium containing basal culture medium, FBS, and dimethyl sulfoxide (DMSO) at a volume ratio of 5:4:1. The cell suspension was aliquoted into cryovials and sequentially stored at 4 °C for 30 min, −20 °C for 2 h, and −80 °C for 24 h before transfer to liquid nitrogen for long-term storage.

### 2.3. Characterization of TSCs

The marker panel was selected to provide complementary phenotypic evidence of the tendon-derived cells. CD44 and CD90 were assessed as mesenchymal stromal cell-associated surface markers, while CD34 was examined as an additional marker relevant to the characterization of the cell population. Nucleostemin (NS), SSEA-4, and Oct-3 were assessed as stem cell-associated markers, and ScxA, Tnmd, and Col I were examined as tendon-associated markers, based on previous studies of TSC characterization [19,20,21]. Information on the antibodies used for these markers, including their manufacturers, catalog numbers, and working dilutions, is provided in Supplementary Table S3.

The expression of stem cell-associated and tendon-associated markers was assessed by flow cytometry (FC500 flow cytometer, Beckman Coulter, Indianapolis, IN, USA) and immunofluorescence staining. For flow cytometry, TSCs were collected, washed with PBS, adjusted to 1 × 10^6^ cells/mL, and incubated with anti-CD44 and anti-CD90 antibodies in PBS containing 2% FBS for 20 min in the dark. After washing twice, the cells were analyzed by flow cytometry using appropriate unstained and single-stained controls. The main cell population was visualized using forward scatter area (FSC-A) versus side scatter area (SSC-A), and the distribution of singlet events was assessed using FSC-A versus forward scatter height (FSC-H). Unstained cells served as negative controls for evaluating background fluorescence in the original flow cytometric analysis. Immunofluorescence staining was used to assess CD34, NS, SSEA-4, Oct-3, ScxA, Tnmd, and Col I. TSCs were seeded on coverslips and fixed with cold acetone for 15 min after cell attachment. Following permeabilization with Triton X-100 and treatment with 3% hydrogen peroxide, the cells were blocked with 5% BSA and incubated overnight at 4 °C with the corresponding primary antibodies. After incubation with fluorescent secondary antibodies and DAPI staining, samples were examined using a fluorescence microscope (Olympus BX43, Olympus, Tokyo, Japan). Primary antibody dilutions were 1:120 for CD34, 1:150 for NS, 1:150 for SSEA-4, 1:100 for Oct-3, 1:120 for ScxA, 1:100 for Tnmd, and 1:100 for Col I.

Clonogenic capacity was evaluated using a colony formation assay with P3 TSCs. TSCs were seeded at 100 or 250 cells/dish, with three replicate dishes per seeding density, and cultured for approximately 7 days, with half of the culture medium replaced every 3 days. Colonies containing more than 50 cells were considered established colonies. After fixation with 4% paraformaldehyde for 30–60 min, the cells were stained with 0.1% crystal violet for 10–20 min. Colony images were acquired and analyzed using ImageJ 1.54g version, and colonies measuring 0.3–1 mm in diameter were counted. Colony formation efficiency was calculated as the number of colonies divided by the number of cells seeded × 100%.

As part of the initial characterization of TSCs, their multilineage differentiation potential was evaluated by osteogenic, chondrogenic, and adipogenic induction. For osteogenic differentiation, TSCs were seeded on 0.1% gelatin-coated 12-well plates at 2 × 10^4^ cells/cm^2^ and cultured to 60–70% confluence. Osteogenic differentiation was induced using a rat tendon stem cell osteogenic differentiation kit (Haixing Biotechnology, Suzhou, China; Cat. No. TSGU-D101) according to the manufacturer’s instructions. After 14–21 days, cells were fixed with 4% paraformaldehyde for 30–60 min and stained with Alizarin Red. Osteogenic differentiation was assessed by Alizarin Red-positive calcium deposits. For chondrogenic differentiation, 3 × 10^5^ TSCs were pelleted and cultured in chondrogenic differentiation medium using a rat tendon stem cell chondrogenic differentiation kit (Haixing Biotechnology, Suzhou, China; Cat. No. TSGU-D203). The induction medium was changed every 2 days, and after approximately 21 days, the pellets were fixed with 4% paraformaldehyde, paraffin-embedded, sectioned, and stained with Alcian Blue. Chondrogenic differentiation was assessed by Alcian Blue-positive staining of acidic mucopolysaccharides. For adipogenic differentiation, TSCs were seeded on 0.1% gelatin-coated 12-well plates at 2 × 10^4^ cells/cm^2^ and cultured to 90–100% confluence. Adipogenic induction and maintenance media were applied in alternating cycles of 3 days of induction medium followed by 1 day of maintenance medium for 14–21 days. Cells were then fixed with 4% neutral buffered formaldehyde for 30–60 min and stained with Oil Red O. Adipogenic differentiation was assessed by the formation of Oil Red O-positive lipid droplets.

### 2.4. Preparation and Characterization of PRP

PRP was prepared using a two-step centrifugation protocol. Under anesthesia with 5% chloral hydrate (350 mg/kg), approximately 5 mL of blood was collected from the right ventricle of each rat into sodium citrate-containing anticoagulant tubes and maintained at 4 °C until processing. Blood samples from the 10 rats were processed separately throughout PRP preparation. Platelet counts in whole blood and PRP were determined manually using a hemocytometer (Model 177-112C, WATSON, Tokyo, Japan), with a 20-μL aliquot used for each measurement. The blood was first centrifuged at 250× *g* for 10 min, and the plasma and platelet-enriched fractions were collected and subjected to a second centrifugation at 1000× *g* for 10 min. After the second centrifugation, the platelet-enriched fraction was collected while the upper plasma fraction and residual erythrocytes were discarded. The platelet enrichment ratio was calculated as the platelet count in PRP divided by that in whole blood. The prepared PRP was immediately used for PRP-EV isolation. No exogenous platelet activator was added before EV isolation.

### 2.5. Preparation and Characterization of PRP-EVs

PRP-EVs were isolated from freshly prepared PRP by differential ultracentrifugation. Each PRP sample was first centrifuged at 2000× *g* for 30 min at 4 °C, followed by centrifugation of the supernatant at 10,000× *g* for 45 min at 4 °C. The resulting supernatant was filtered through a 0.45-μm membrane and ultracentrifuged at 100,000× *g* for 70 min at 4 °C. The pellet was resuspended in 10 mL pre-cooled PBS and subjected to a second ultracentrifugation at 100,000× *g* for 70 min at 4 °C. The final pellet from each preparation was resuspended in 100 μL pre-cooled PBS. The 10 individual PRP-EV preparations were subsequently pooled, aliquoted, and stored at −80 °C until characterization or use. Individual aliquots were thawed as needed and were not subjected to repeated freeze–thaw cycles. All characterization procedures described below were performed using this pooled preparation.

The ultrastructure of PRP-EVs was examined by transmission electron microscopy (TEM). Briefly, 20 μL of freshly prepared PRP-EV suspension was loaded onto a carbon-coated copper grid for 3–5 min, followed by negative staining with 2% phosphotungstic acid for 1–2 min. After air-drying at room temperature, the samples were examined by TEM and representative images were obtained. Multiple fields were examined, and one representative image was selected to illustrate the morphology of the pooled PRP-EV preparation.

The particle-size distribution of PRP-EVs was measured by dynamic light scattering (DLS) using a Zetasizer Nano-ZS 90 (nanoparticle size and zeta potential analyzer, Malvern Panalytical, Malvern, UK) at a scattering angle of 90°. A 100 μL aliquot of the pooled PRP-EV suspension was diluted with 2–3 mL PBS and mixed thoroughly. The diluted sample was loaded into the measurement cell and equilibrated at 25 °C for 5 min before measurement. Three consecutive technical measurements were performed on the same sample in the measurement cell, and the particle-size distribution data were generated using the instrument software.

Western blotting (WB) was performed to assess the EV-associated proteins TSG101, CD9, and CD81 in the pooled PRP-EV preparation. Before WB analysis, total protein concentration was determined using a bicinchoninic acid (BCA) protein assay kit (Servicebio, Wuhan, China; Cat. No. G2026). Bovine serum albumin standards ranging from 0 to 500 μg/mL were used to generate a standard curve. For each measurement, 20 μL of standard or appropriately diluted sample was mixed with 200 μL of BCA working reagent, prepared by mixing the BCA reagent and copper sulfate solution at a 50:1 volume ratio. After incubation at 37 °C for 30 min, absorbance was measured at 562 nm using a microplate reader. Sample protein concentrations were calculated from the standard curve and corrected for the dilution factor. For WB analysis, 20 μg of total protein per sample was separated on 10% precast gels and transferred onto PVDF membranes.

After blocking with protein-free rapid blocking buffer for 1 h at room temperature, the membranes were incubated overnight at 4 °C with primary antibodies against TSG101 (1:1000), CD9 (1:1000), CD81 (1:1000), and β-actin (1:1600). After washing with Tris-buffered saline with Tween 20 (TBST), the membranes were incubated with HRP-conjugated secondary antibodies (1:10,000) for 1 h at room temperature. Protein bands were visualized using an enhanced chemiluminescence (ECL) detection system and quantified using ImageJ 1.54g version. β-actin was included as a loading control. The manufacturers, catalogue numbers, working dilutions, and expected molecular weights of the antibodies are provided in the Supplementary Table S1.

### 2.6. PRP-EV Treatment of TSCs

PRP-EVs stored at −80 °C were thawed at 4 °C, and the total protein concentration of the pooled preparation was determined using a BCA assay. Based on the measured total protein concentration and concentrations reported in previous studies [16,22,23], the preparation was diluted with complete culture medium to obtain final PRP-EV total protein concentrations of 5, 20, and 40 μg/mL.

Cryovials containing TSCs were retrieved from liquid nitrogen and rapidly thawed in a 37 °C water bath. The thawed cell suspension was immediately transferred into a 15 mL centrifuge tube containing 5 mL of prewarmed complete medium and centrifuged at 1500 rpm for 5 min. The cells were resuspended in complete medium and cultured in T25 flasks at 37 °C in a humidified atmosphere containing 5% CO_2_ for 24 h. When TSCs reached approximately 80% confluence, the cells were detached with 1 mL trypsin-EDTA and collected by centrifugation at 1500 rpm for 5 min. The cells were resuspended in fresh complete medium, and cell density was adjusted to 2 × 10^4^ cells/cm^2^ for seeding into 96-well plates or T25 flasks. The cells were pre-cultured for 24 h before subsequent experiments.

After cell attachment, TSCs were divided into four groups according to the final PRP-EV concentration in the culture medium: Control (0 μg/mL), 5 μg/mL, 20 μg/mL, and 40 μg/mL. The same grouping was applied to TSCs cultured in 96-well plates and T25 flasks. Each well of the 96-well plates and each T25 flask received 100 μL and 5 mL of the corresponding treatment medium, respectively. The culture medium was replaced every 2 days throughout the treatment period.

For CCK-8 signal assessment, TSCs cultured in 96-well plates were treated with PRP-EVs for 1, 3, or 5 days, with three wells per group. At each time point, CCK-8 solution (10 μL) was added to each well, and the cells were incubated for 2 h at 37 °C. Absorbance was measured at 450 nm using a microplate reader. The same wells were measured at all three time points. To correct for background absorbance, the mean absorbance of blank wells containing culture medium and CCK-8 reagent but no cells was calculated for each time point and subtracted from the OD450 value of each corresponding sample well. The resulting blank-corrected OD450 values were used for statistical analysis.

For molecular analyses of tendon- and lineage-associated markers, TSCs cultured in T25 flasks were treated with PRP-EVs for 6 days and subsequently collected for quantitative real-time PCR (qPCR) and WB analysis.

### 2.7. Analysis of Tendon- and Lineage-Associated Markers in TSCs

qPCR and Western blotting were performed to assess the mRNA and protein expression levels of tendon-associated markers and lineage-associated markers in TSCs after PRP-EV treatment.

For qPCR analysis, gene-specific primers were designed and synthesized by Sangon Biotech (Shanghai, China). The forward and reverse primer sequences, GenBank accession numbers, and expected amplicon lengths for all target genes and the reference gene are provided in Supplementary Table S2. β-actin (Actb; GenBank accession No. NM_031144.3) was used as the reference gene for normalization. Total RNA was extracted using TRIzol reagent. Approximately 1 × 10^6^–10^7^ TSCs were lysed in 1 mL TRIzol, followed by phase separation with 0.2 mL chloroform and centrifugation at 12,000 rpm for 15 min at 4 °C. The aqueous phase was collected and mixed with 0.5 mL isopropanol for RNA precipitation. The RNA pellet was washed with 1 mL of 75% ethanol, air-dried, and dissolved in DEPC-treated water. RNA concentration and purity were assessed using a DeNovix spectrophotometer (DS-11 microvolume UV–Vis spectrophotometer, DeNovix, Wilmington, DE, USA) Samples with OD260/OD280 ratios of 1.7–2.0 and OD260/OD230 ratios of 2.2–2.5 were used for subsequent analysis.

Reverse transcription was performed using the HiFiScript Fast Genomic DNA Removal and cDNA First-Strand Synthesis Kit according to the manufacturer’s instructions. Genomic DNA was removed at 42 °C for 2 min, followed by reverse transcription at 42 °C for 15 min and enzyme inactivation at 85 °C for 5 min. qPCR was performed using MonAmp™ ChemoHS qPCR Mix (Monad Biotech, Shanghai, China; Cat. No. MQ00401S) on a Bio-Rad real-time PCR system (CFX Connect real-time PCR system, Bio-Rad, Hercules, CA, USA). The composition of the 20-μL qPCR reaction mixture is provided in Supplementary Table S5. Each cDNA sample was analyzed in triplicate using the corresponding target-gene or reference-gene primers. The amplification protocol consisted of an initial denaturation at 95 °C for 15 min, followed by 40 cycles of 95 °C for 10 s, 53–58 °C for 30 s, and 72 °C for 30 s. Melting curve analysis was subsequently performed from 60 to 95 °C, with a temperature increase of 0.3 °C every 15 s. The mean value of the three technical replicates was used for subsequent analysis.

The relative mRNA expression levels were calculated using the 2^−ΔΔCT^ method, with the first sample in the Control group serving as the calibrator. Relative expression (*F*) was calculated as follows:

*F* = 2^−ΔΔ*CT*^

where

ΔΔ*C_t_* = (*C_t_*_,target_ − *C_t_*_,reference_)_treatment_ − (*C_t_*_,target_ − *C_t_*_,reference_)_control_.

For WB analysis, TSCs were lysed using radio immunoprecipitation assay (RIPA) buffer containing phenylmethylsulfonyl fluoride (PMSF). Approximately 250 μL of RIPA lysis buffer containing 2.5 μL of PMSF protease inhibitor was added per 1 × 10^6^ cells. Cells were lysed by ultrasonic disruption on ice for 1 min and centrifuged at 12,000 rpm for 10 min at 4 °C. The supernatant was collected, and protein concentration was determined using a BCA assay. Equal amounts of total protein (20 μg) were separated on 10% precast gels, transferred onto PVDF membranes, and blocked with protein-free rapid blocking solution for 1 h at room temperature. The membranes were incubated overnight at 4 °C with primary antibodies against ScxA (1:1200), Tnmd (1:1000), Col I (1:1000), Osx (1:1000), RUNX2 (1:1000), SOX9 (1:1000), PPARγ (1:1000), and β-actin (1:1600). After washing with TBST, the membranes were incubated with the appropriate HRP-conjugated secondary antibody (goat anti-rabbit, ZSGB-BIO, Beijing, China, Cat. No. ZB2301, 1:10,000; or goat anti-mouse, ZSGB-BIO, Beijing, China, Cat. No. ZB2305, 1:10,000) for 1 h at room temperature and subsequently washed with TBST. Protein bands were visualized using an ECL detection system, and band intensities were quantified using ImageJ 1.54g version. For each target protein, samples from all four treatment groups and the corresponding β-actin signals were analyzed on the same membrane. Target protein band intensities were normalized to the corresponding β-actin band intensities, and the normalized values were used for statistical analysis. Details of all primary and secondary antibodies are provided in Supplementary Table S1, including their targets or specificities, host species, manufacturers, catalogue numbers, and working dilutions, together with the expected molecular weights of the primary targets.

### 2.8. Statistical Analysis

Statistical analyses were performed using SPSS Statistics version 22.0 for Windows (IBM Corp., Armonk, NY, USA). Statistical analyses were performed using SPSS Statistics version 22.0 for Windows (IBM Corp., Armonk, NY, USA). Data are presented as mean ± standard deviation (SD). Normality was assessed using the Shapiro–Wilk test, and homogeneity of variance was assessed using Levene’s test. No significant departures from these assumptions were detected.

For CCK-8 analysis, three wells per group were included, and the same wells were measured repeatedly on days 1, 3, and 5. Blank-corrected OD450 values were used for statistical analysis. Changes in the CCK-8 signal over time were analyzed using two-way repeated-measures analysis of variance (ANOVA), with treatment group as the between-subject factor and time as the within-subject factor. Mauchly’s test was used to assess the assumption of sphericity. As the sphericity assumption was met, the sphericity-assumed results were used without Greenhouse–Geisser correction. When a significant group × time interaction was observed, simple effects analyses were performed to examine group differences at each time point and time differences within each group. Bonferroni correction was applied to pairwise comparisons. Partial eta squared (ηp^2^) was used to quantify the effect sizes of the group, time, and interaction effects.

For qPCR and Western blot (WB) analyses, three separately treated T25 flasks per group were included. All treatment groups were derived from a single pooled TSC preparation. Relative mRNA expression values calculated using the 2^−ΔΔCt^ method and protein expression values normalized to β-actin were analyzed separately for each marker using one-way ANOVA. Bonferroni correction was applied to the overall ANOVA *p* values across the seven markers assessed by qPCR and the seven markers assessed by WB, with each assay treated as a separate family of tests. Bonferroni-adjusted pairwise comparisons were used to evaluate differences among the four treatment groups within each marker. Mean differences and their corresponding 95% confidence intervals (CIs) were reported for pairwise comparisons.

## 3. Results

### 3.1. Morphological Observation of TSCs

**Figure 1:**
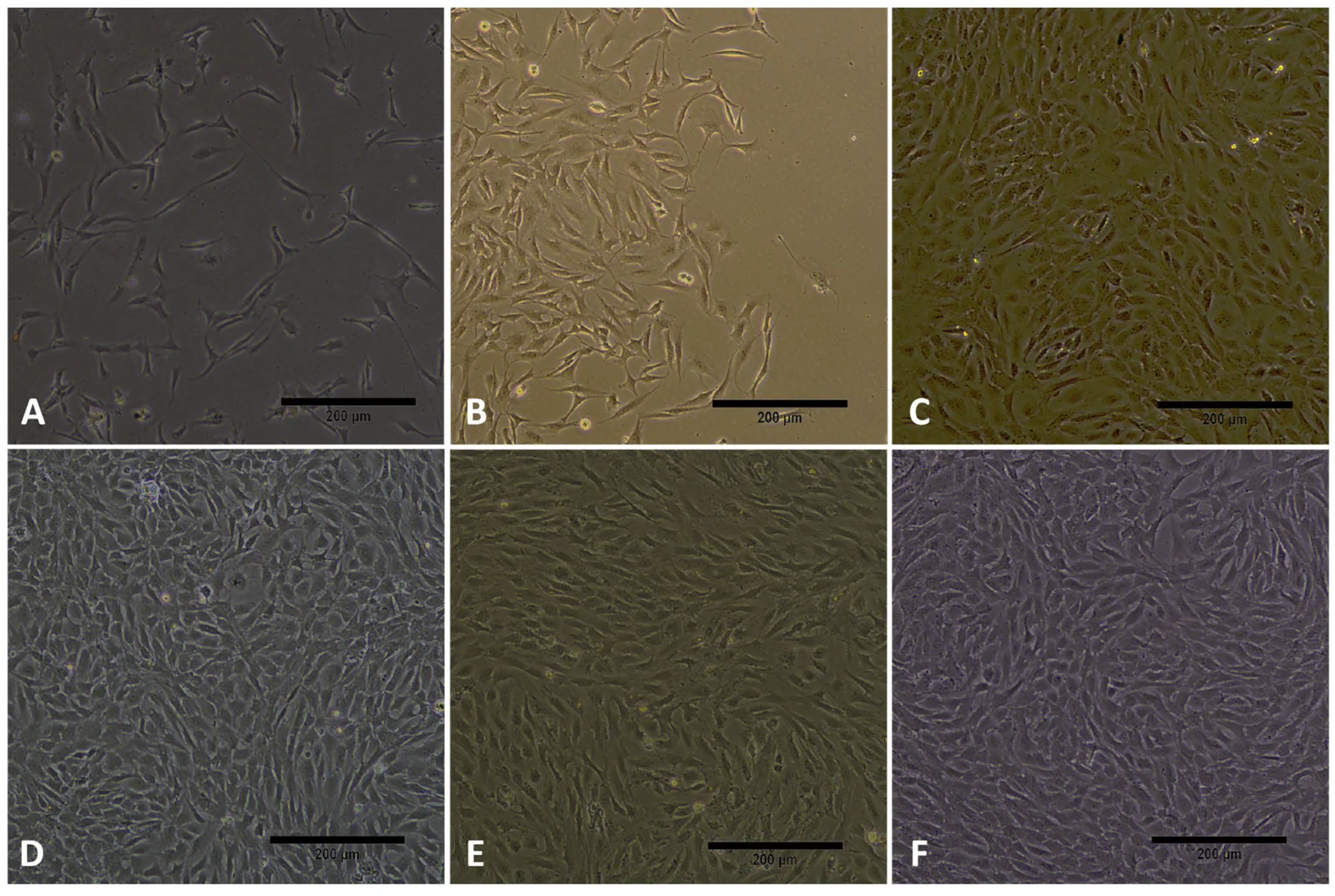
Microscopic observation of TSC morphology and cell density during culture (100×). Note: (**A**–**C**) show P0 TSCs cultured for 3, 5, and 8 days, respectively; (**D**–**F**) show TSCs at passages P1, P2, and P3, respectively. Representative images are shown from the single pooled TSC preparation derived from three rats.

As shown in Figure 1, cells isolated from rat Achilles tendon tissue by enzymatic digestion exhibited a cobblestone-like morphology during in vitro culture. Cell density gradually increased from day 3 to day 8, and the cells approached confluence by day 8. The cells maintained consistent morphology during subsequent passages from P1 to P3.

### 3.2. Phenotypic Characterization of TSCs

**Figure 2:**
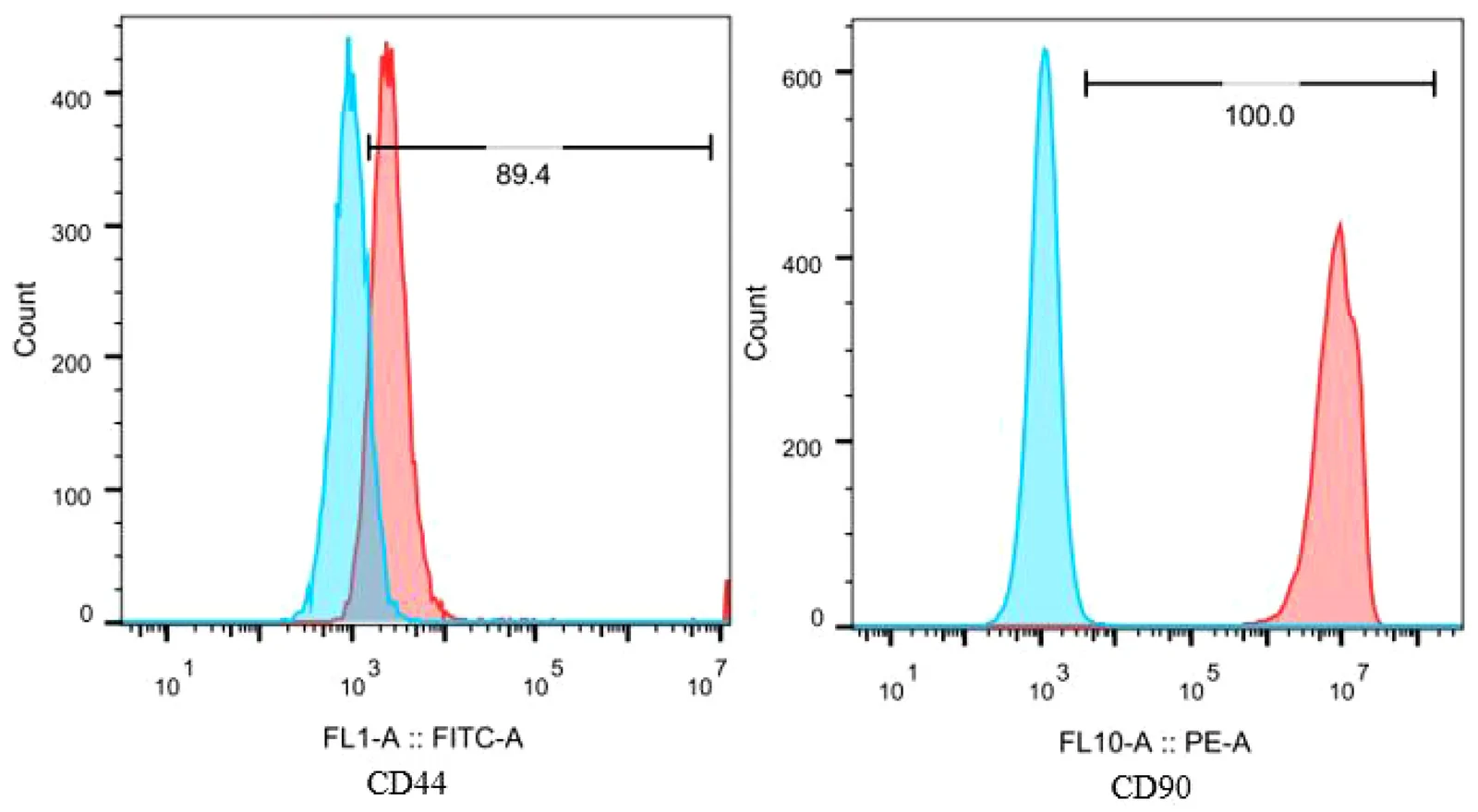
Flow cytometric characterization of CD44 and CD90 expression on the surface of TSCs. The analysis was performed using a single pooled TSC preparation derived from three rats (one pooled preparation, *n* = 1). Unstained cells served as negative controls. The reported positive-cell proportions were 89.4% for CD44 and 100% for CD90.

**Figure 3:**
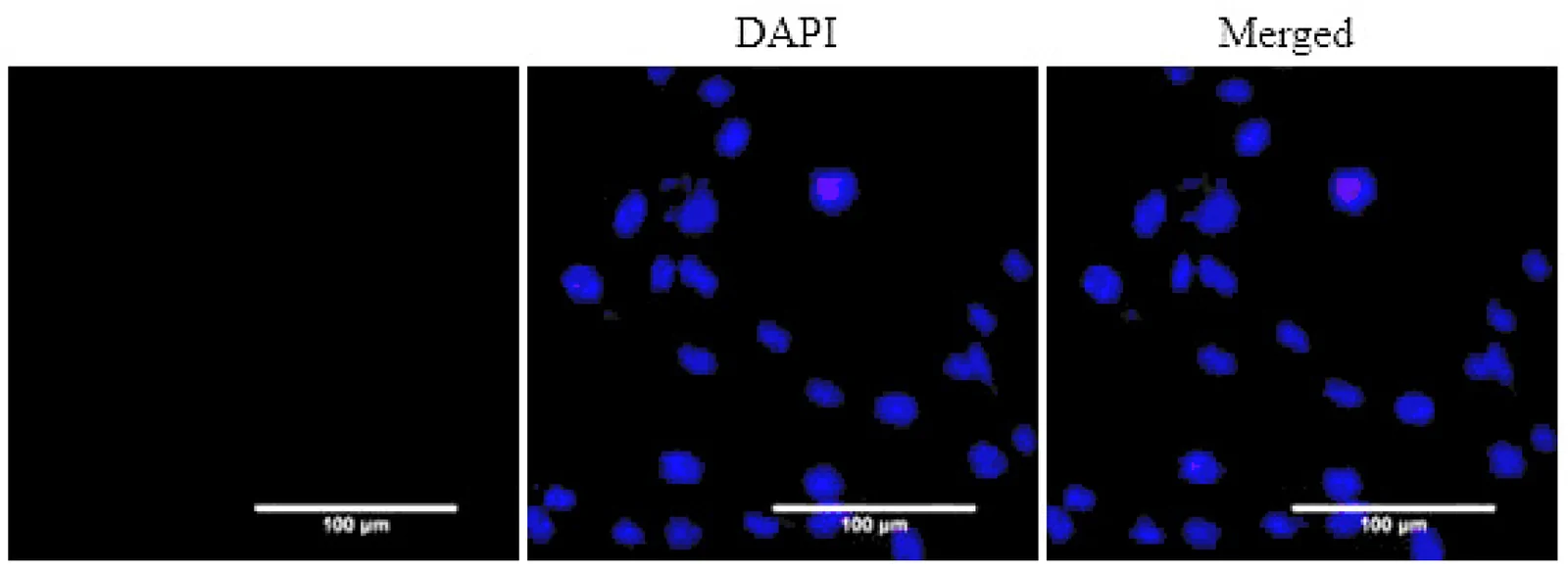
Representative immunofluorescence image showing no detectable CD34 expression in TSCs (200×). Images were obtained from the single pooled TSC preparation derived from three rats (*n* = 1 pooled preparation).

Flow cytometry was performed to assess the expression of the mesenchymal stem cell-associated surface markers CD44 and CD90 in TSCs. The positive rates of CD44 and CD90 were 89.4% and 100%, respectively (Figure 2). Immunofluorescence staining showed no detectable CD34 expression in TSCs (Figure 3). Representative gating plots illustrating the identification of the main cell population and the assessment of singlet events are provided in Supplementary Figure S4.

**Figure 4:**
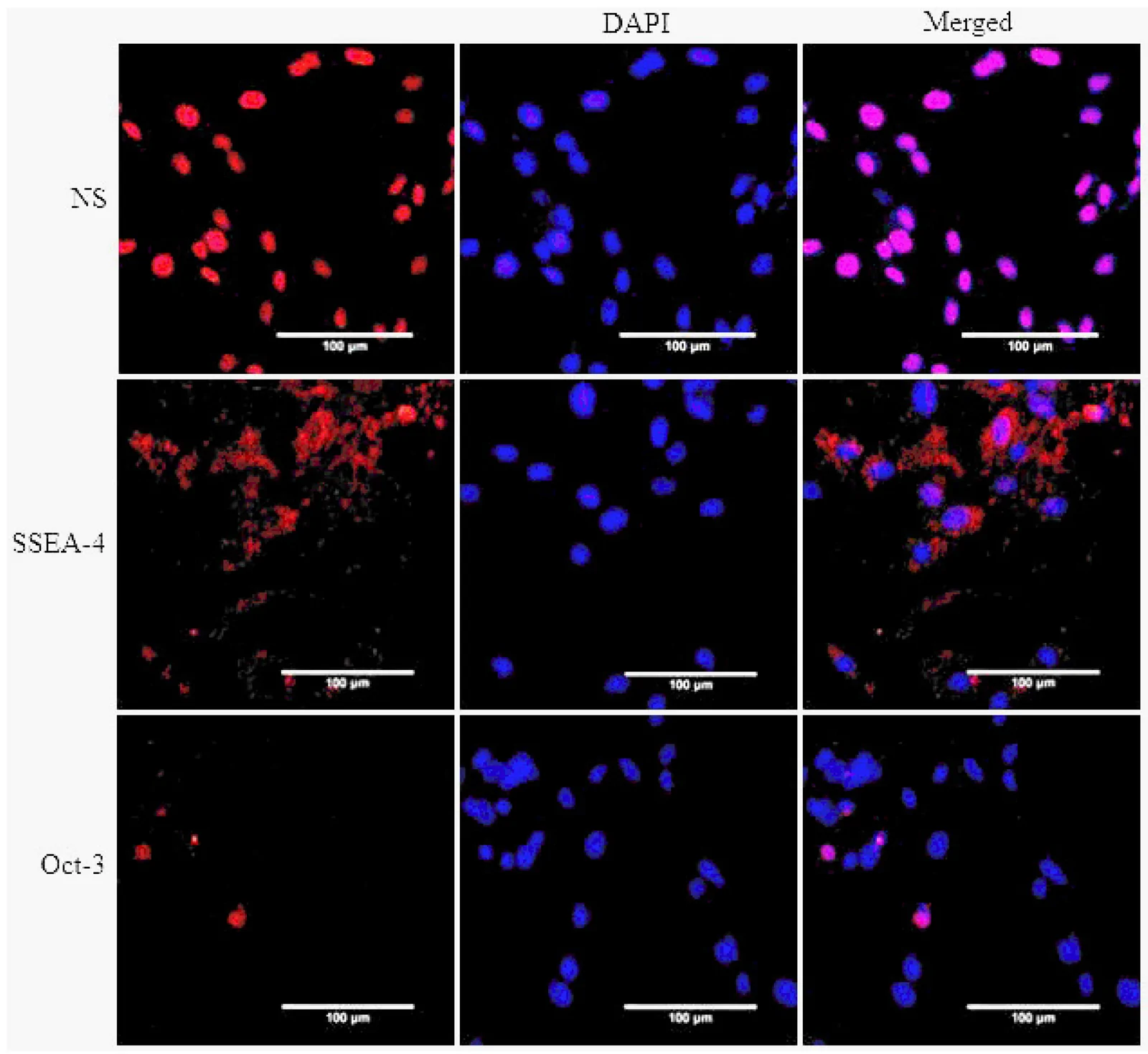
Representative immunofluorescence images showing positive expression of the stem cell-associated markers NS, SSEA-4, and Oct-3 in TSCs (200×). Images were obtained from the single pooled TSC preparation derived from three rats (*n* = 1 pooled preparation).

**Figure 5:**
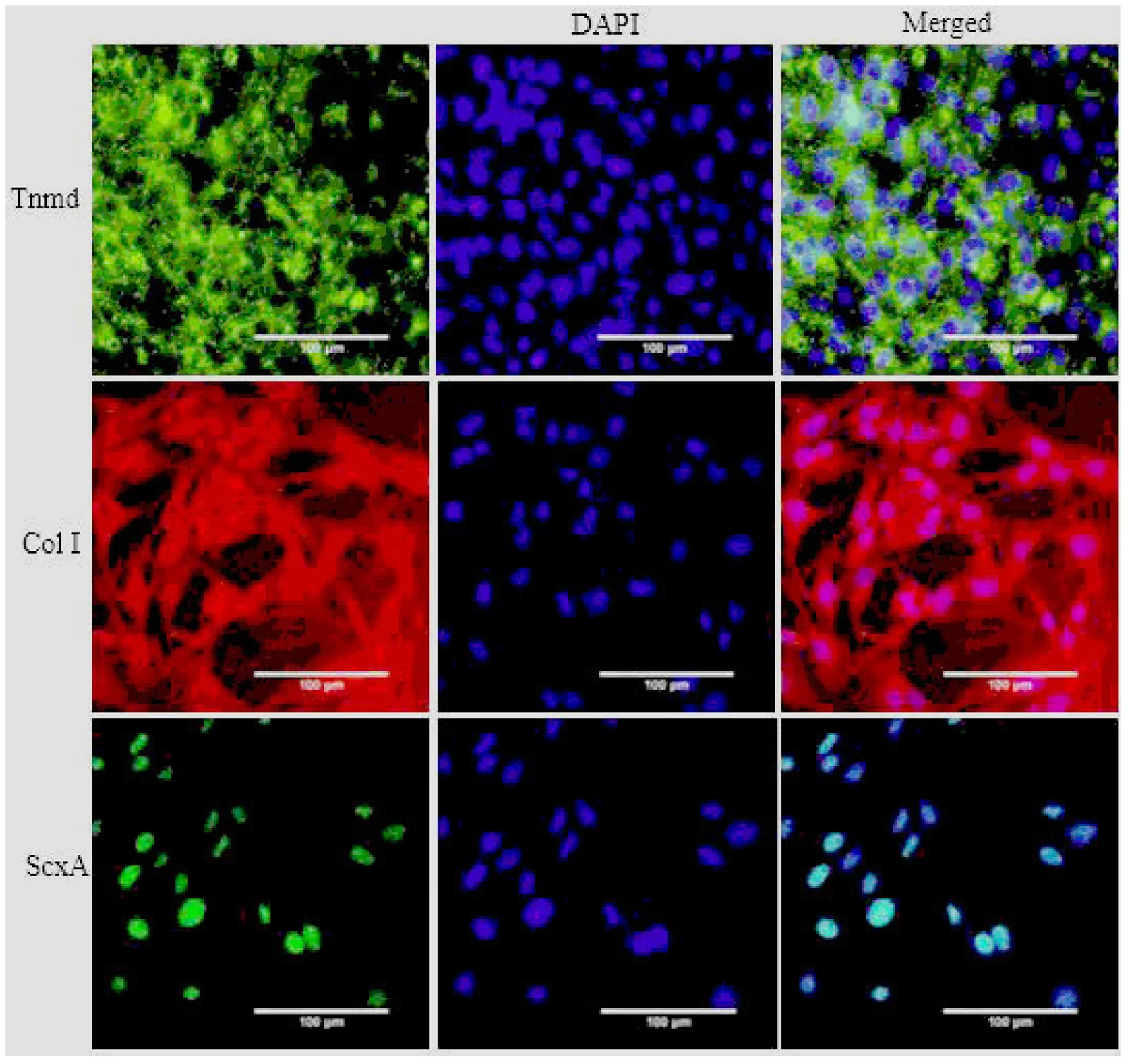
Representative immunofluorescence images showing positive expression of the tendon-associated markers Tnmd, Col I, and ScxA in TSCs (200×). Images were obtained from the single pooled TSC preparation derived from three rats (*n* = 1 pooled preparation).

Immunofluorescence staining further showed positive expression of the stem cell-associated markers NS, SSEA-4, and Oct-3 in TSCs (Figure 4). In addition, the tendon-associated markers ScxA, Tnmd, and Col I were positively expressed in TSCs (Figure 5).

### 3.3. Colony Formation Capacity of TSCs

**Figure 6:**
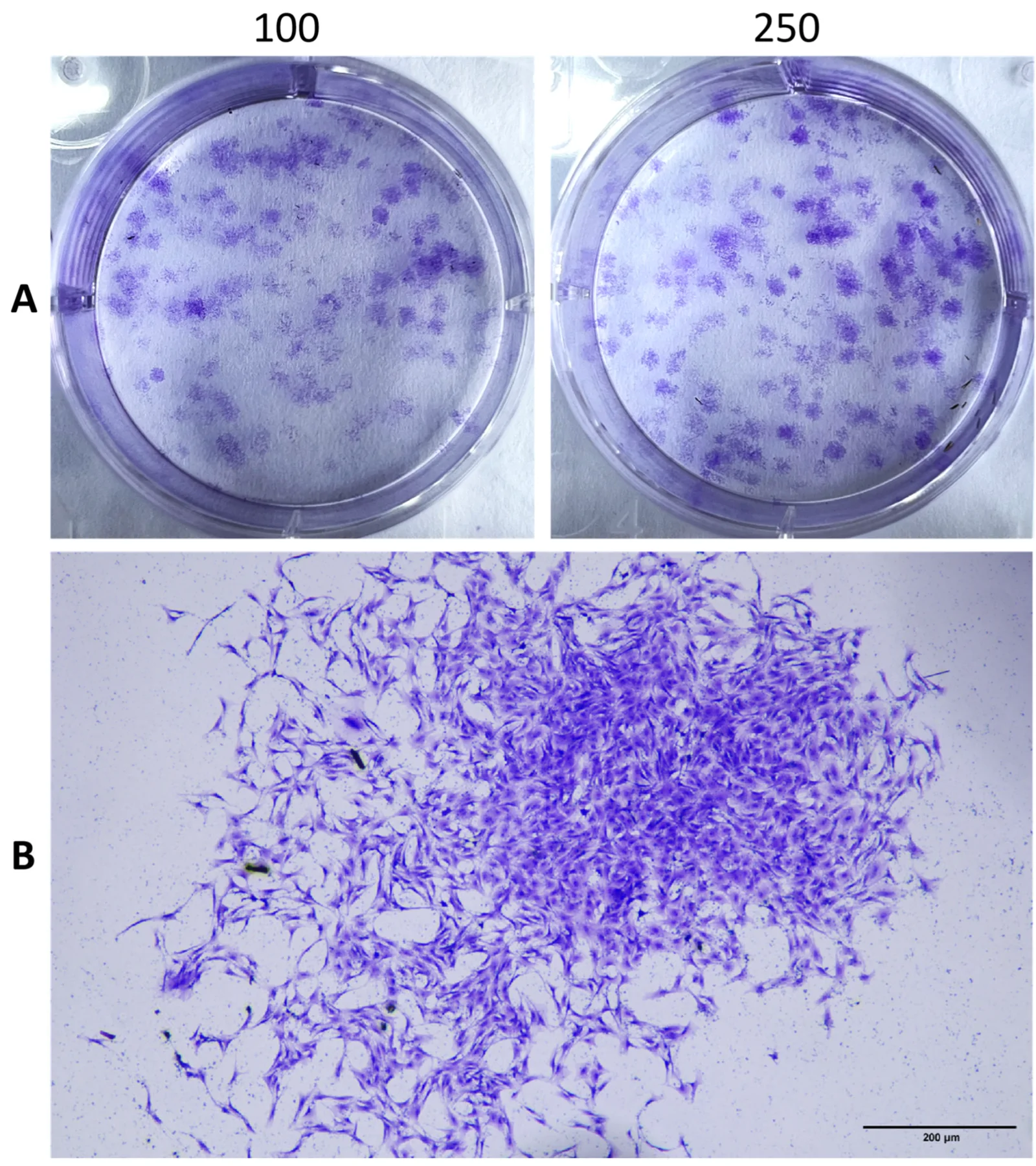
Colony formation and morphological characteristics of TSCs. Note: (**A**), representative colony formation images of TSCs seeded at 100 and 250 cells/dish, respectively; (**B**), representative colony morphology (100×). Images were obtained using TSCs from the single pooled preparation derived from three rats.

TSCs formed visible colonies during in vitro culture when seeded at 100 or 250 cells/dish (Figure 6). The corresponding colony formation efficiencies are presented in Table 1. These results indicated that TSCs possessed clonogenic capacity under the present culture conditions.

### 3.4. Characterization of the Multilineage Differentiation Potential of TSCs

As part of the initial characterization of TSCs, their multilineage differentiation potential was assessed through osteogenic, chondrogenic, and adipogenic induction.

**Figure 7:**
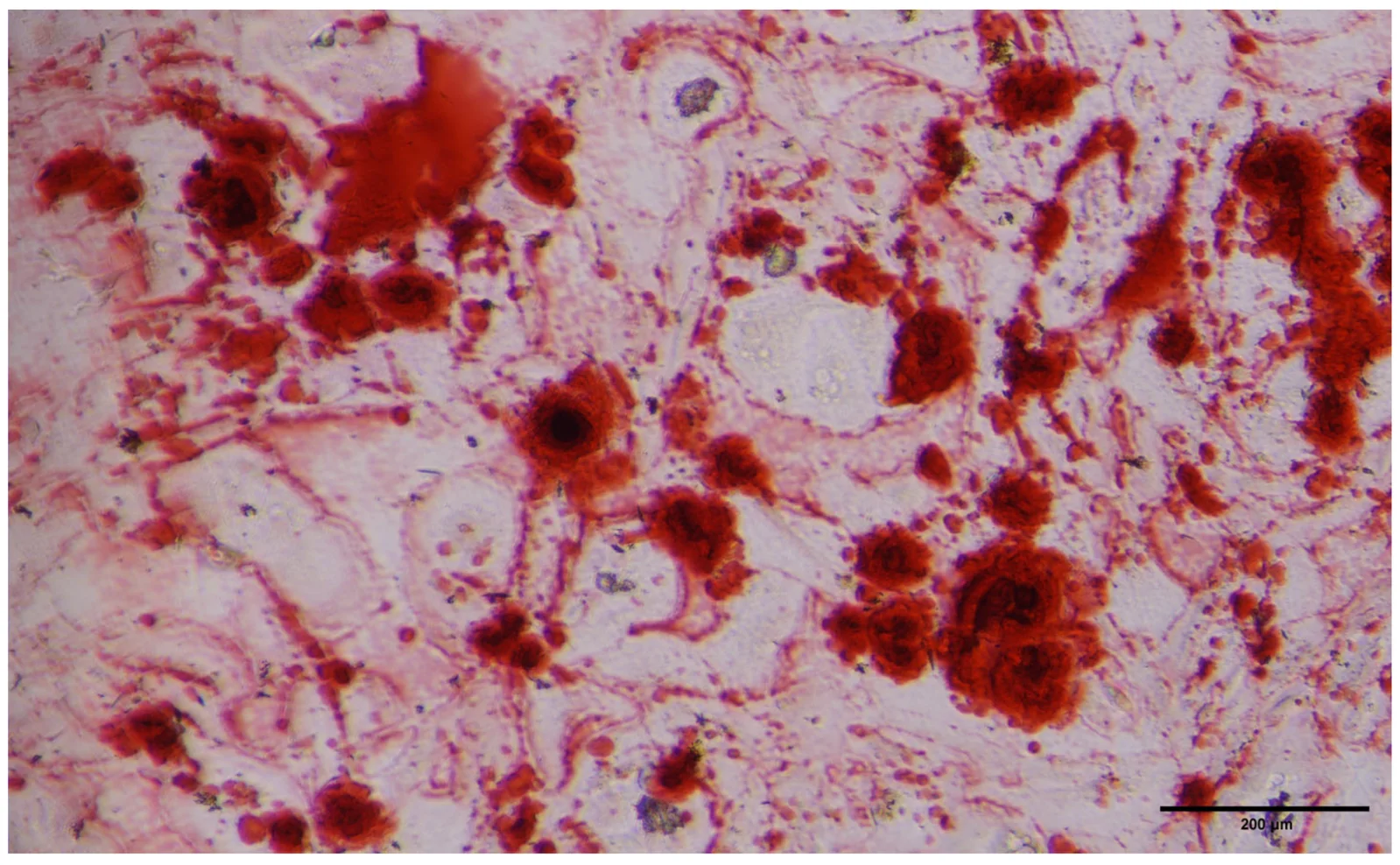
Characterization of osteogenic differentiation of TSCs (100×). Representative image from the single pooled TSC preparation derived from three rats.

During osteogenic induction, cell density increased markedly after 8–10 days, with localized mineralized deposition becoming apparent. After 17–21 days of induction, mineralized nodules were observed, and the cells showed positive Alizarin Red staining (Figure 7), supporting osteogenic differentiation.

**Figure 8:**
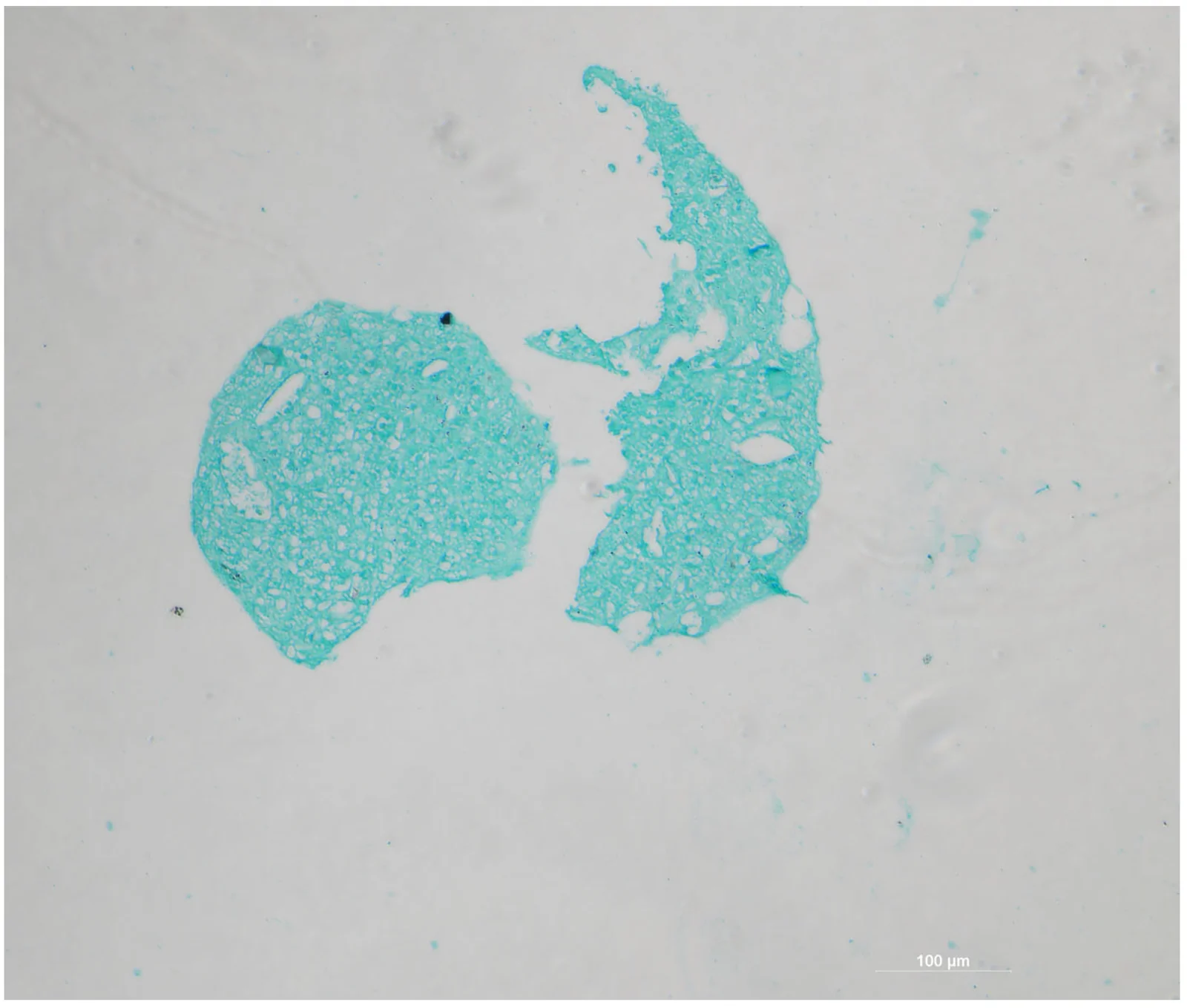
Characterization of chondrogenic differentiation of TSCs (200×). Representative image from the single pooled TSC preparation derived from three rats.

During chondrogenic induction, TSCs began to aggregate and form cell pellets after 24–48 h. After approximately 21 days of induction, the pellets became distinctly spherical with a smooth surface, and Alcian Blue staining was positive (Figure 8), supporting chondrogenic differentiation.

**Figure 9:**
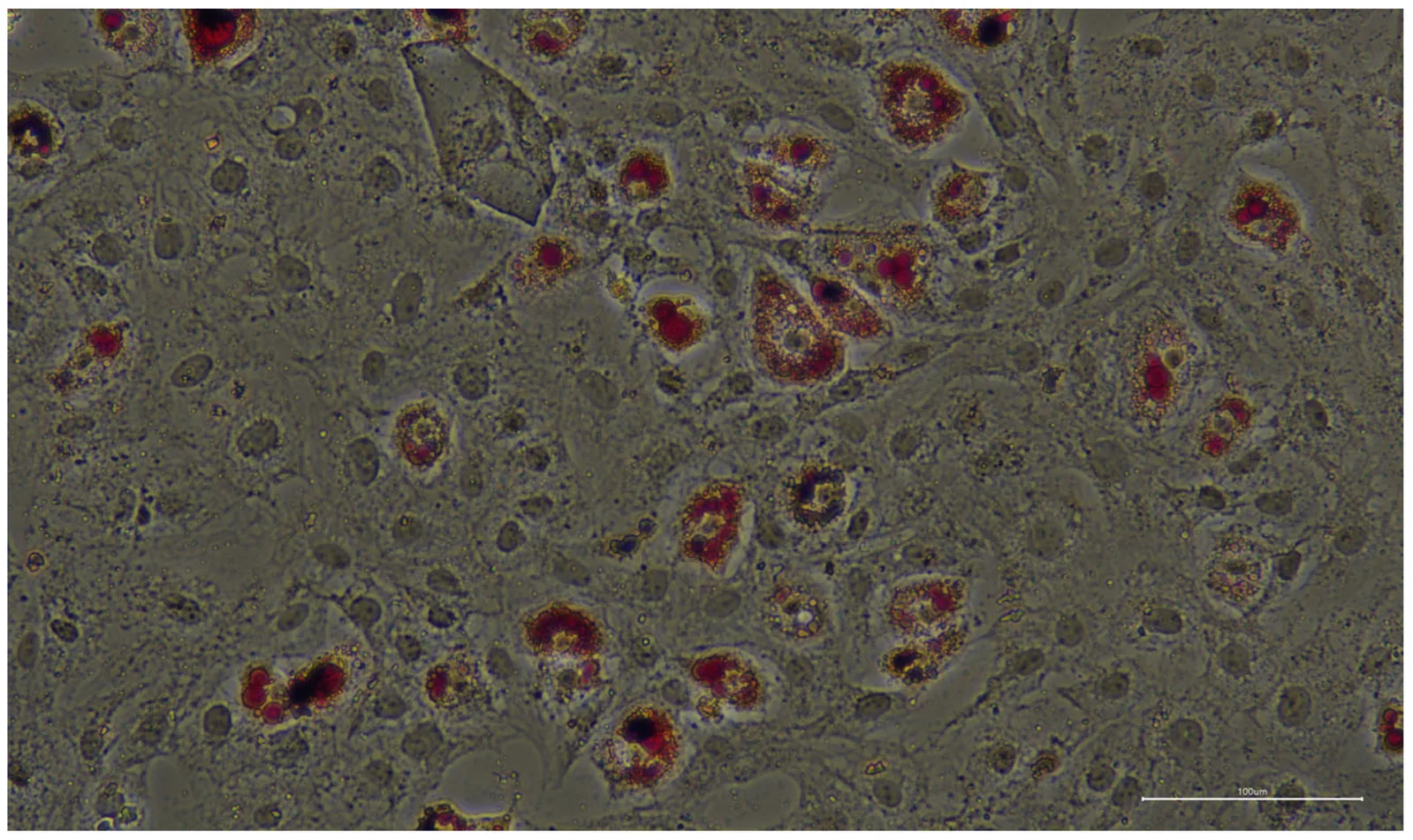
Characterization of adipogenic differentiation of TSCs (200×). Representative image from the single pooled TSC preparation derived from three rats.

During adipogenic induction, small lipid droplets began to appear within some TSCs after 5–6 days. After 14–21 days of induction, the number and size of lipid droplets increased, accompanied by positive Oil Red O staining (Figure 9), supporting adipogenic differentiation.

### 3.5. Characterization of PRP

PRP was prepared from blood collected from the right ventricles of 10 rats. The platelet concentration in PRP from each rat exceeded 1600 × 10^9^/L, and the platelet enrichment ratio was approximately 4 to 5 (Table 2). These results indicated successful platelet enrichment during PRP preparation.

### 3.6. Characterization of PRP-EVs

**Figure 10:**
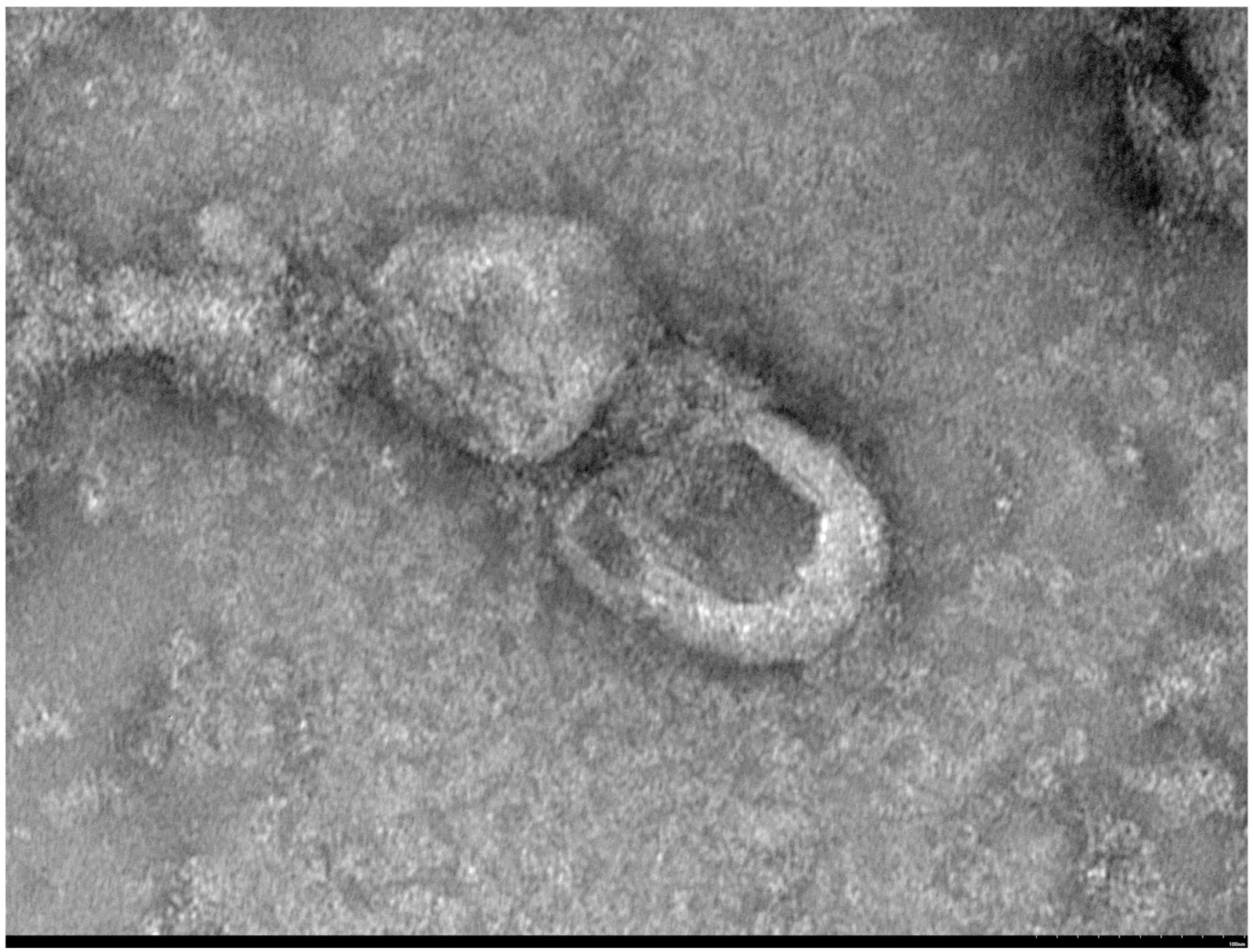
Ultrastructural characteristics of PRP-EVs observed by transmission electron microscopy (60,000×). Representative image of the single pooled PRP-EV preparation (*n* = 1).

TEM was used to examine the ultrastructural characteristics of PRP-EVs. The prepared PRP-EVs exhibited approximately round or oval vesicular structures surrounded by a bilayer lipid membrane (Figure 10).

**Figure 11:**
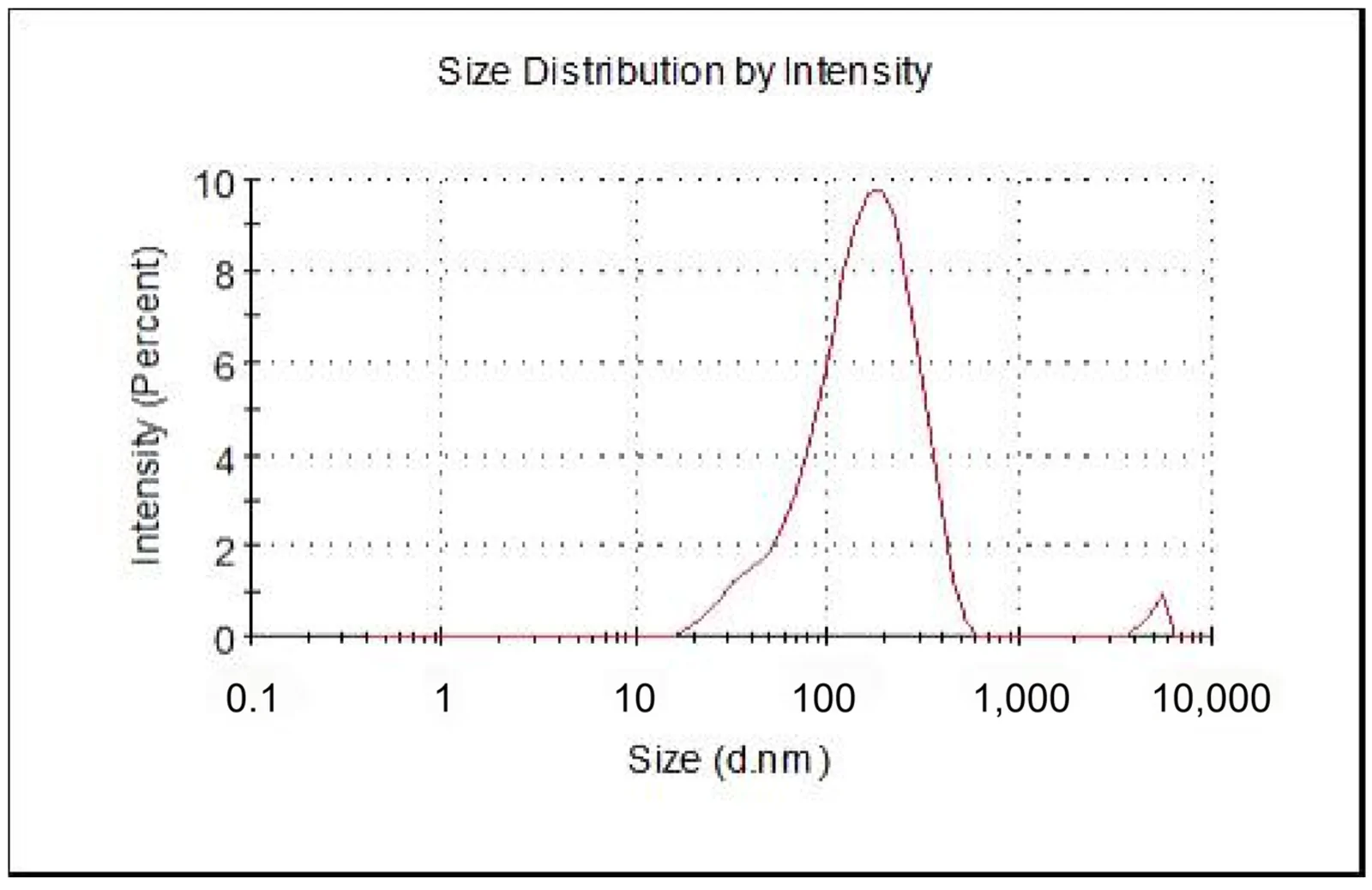
Intensity-based particle-size distribution of PRP-EVs measured by DLS in the single pooled preparation (*n* = 1 pooled preparation; three consecutive technical measurements).

The DLS analysis showed a mean particle diameter of 198.6 ± 94.5 nm. The intensity-based size-distribution plot showed a dominant signal within the approximate 10–1000 nm range and a smaller signal within the approximate 1000–10,000 nm range. The distribution represents relative scattering intensity (Figure 11).

**Figure 12:**
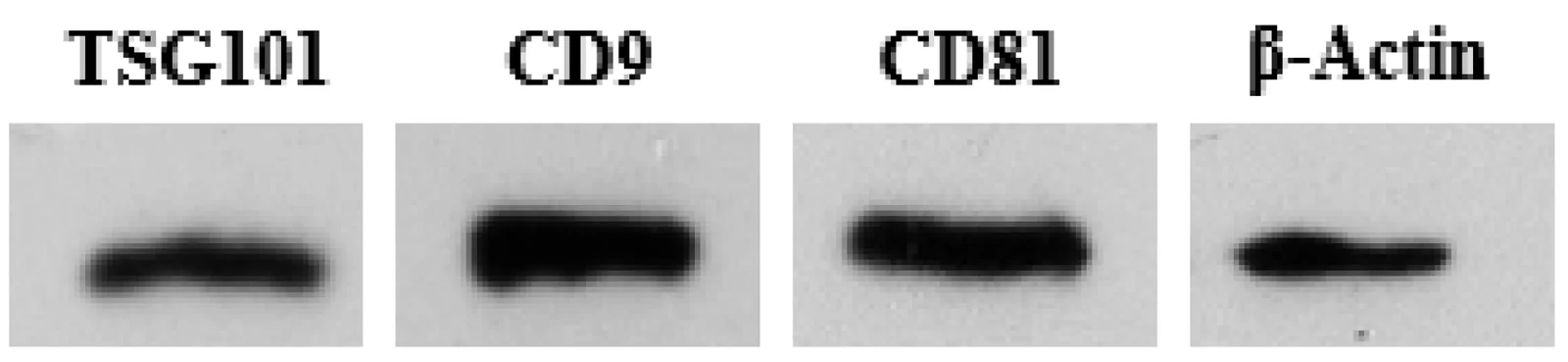
Western blot analysis of the EV-associated proteins TSG101, CD9, and CD81 in the single pooled PRP-EV preparation (*n* = 1 pooled preparation). β-actin was included as a loading control.

WB was performed to assess the EV-associated proteins TSG101, CD9, and CD81 in the pooled PRP-EV preparation. All three proteins were detected (Figure 12). Uncropped WB images are provided in Supplementary Figure S1. Collectively, TEM, DLS, and WB provided complementary evidence of EV-associated morphological and molecular features in the pooled preparation.

### 3.7. Effects of PRP-EVs on CCK-8 Signal in TSCs

**Figure 13:**
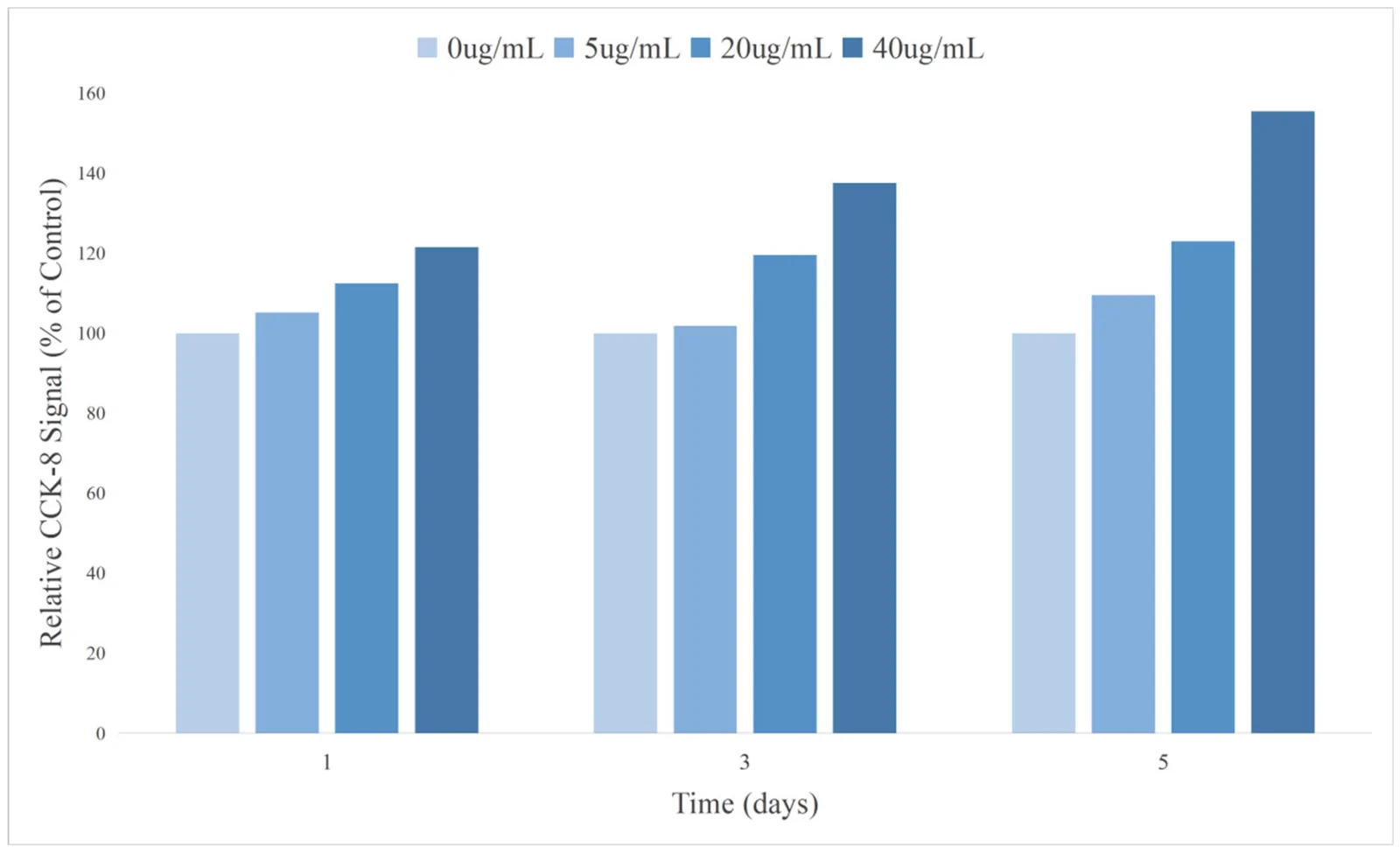
Relative CCK-8 Signal Following PRP-EV Treatment. The mean blank-corrected OD450 value for each treatment group at each time point was expressed as a percentage of the mean value of the corresponding control group, which was set to 100%. Bars represent normalized mean values.

The CCK-8 assay was used to assess changes in the CCK-8 signal in TSCs following treatment with different concentrations of PRP-EVs. Statistical analysis was performed using two-way repeated-measures ANOVA based on blank-corrected OD450 values before normalization. Significant main effects of treatment group and time, as well as a significant treatment group × time interaction, were observed (Table 3). For visualization, the mean CCK-8 signal in each treatment group at each time point was normalized to the mean value of the corresponding control group, which was set to 100%. The resulting relative CCK-8 signals are presented in Figure 13.

Simple effect analysis of time showed no significant changes in the CCK-8 signal across the measured time points in the Control group [F(2, 7) = 0.057, *p* = 0.945, ηp^2^ = 0.016] or the 5 μg/mL PRP-EV group [F(2, 7) = 3.221, *p* = 0.102, ηp^2^ = 0.479]. In contrast, significant time effects were observed in the 20 μg/mL PRP-EV group [F(2, 7) = 8.527, *p* = 0.013, ηp^2^ = 0.709] and the 40 μg/mL PRP-EV group [F(2, 7) = 67.826, *p* < 0.001, ηp^2^ = 0.951]. Further pairwise comparisons showed that the CCK-8 signal in the 20 μg/mL group was significantly higher on day 5 than on day 1. In the 40 μg/mL group, the CCK-8 signal increased significantly from day 1 to day 3 and further increased from day 3 to day 5.

Simple effect analysis of group showed significant differences among PRP-EV concentrations at day 1 [F(3, 8) = 27.927, *p* < 0.001, ηp^2^ = 0.913], day 3 [F(3, 8) = 27.022, *p* < 0.001, ηp^2^ = 0.910], and day 5 [F(3, 8) = 44.355, *p* < 0.001, ηp^2^ = 0.943]. Pairwise comparisons showed that at day 1, the CCK-8 signal in the 20 and 40 μg/mL groups was significantly higher than that in the Control group, and the 40 μg/mL group showed a significantly higher signal than the 5 and 20 μg/mL groups. At day 3, the CCK-8 signal in the 20 and 40 μg/mL groups was significantly higher than that in the Control and 5 μg/mL groups, while the 40 μg/mL group showed a significantly higher signal than the 5 μg/mL group. At day 5, the CCK-8 signal in the 20 and 40 μg/mL groups was significantly higher than that in the Control group, and the 40 μg/mL group showed a significantly higher signal than the 5 and 20 μg/mL groups.

These findings indicate that PRP-EV treatment was associated with increased CCK-8 signal, with the magnitude of the response varying across concentrations and time points. Given that the CCK-8 assay primarily reflects cellular metabolic activity, these results should be interpreted as changes in metabolic activity rather than direct evidence of increased cell proliferation, particularly in light of the limited biological replication.

### 3.8. Effects of PRP-EVs on the Expression of Lineage-Associated Markers in TSCs

#### 3.8.1. Effects of PRP-EVs on the mRNA Expression of Lineage-Associated Markers

qPCR was performed to assess the effects of different concentrations of PRP-EVs on the mRNA expression levels of lineage-associated markers in TSCs. The amplification products of all target genes were within the expected size range of 80–300 bp. The amplification curves showed identifiable exponential phases, and the melting curves exhibited a predominant peak for each assay, supporting amplification specificity (Supplementary Figure S3).

As shown in Table 4 and Table 5, compared with the Control group, the mRNA expression levels of the tendon-associated markers ScxA, Col1a1, and Tnmd were higher in all three PRP-EV-treated groups. However, the differences between the Control and 5 μg/mL groups were not statistically significant for any of these markers, whereas significant increases were observed in the 20 and 40 μg/mL groups. Compared with the 5 μg/mL group, the expression levels of these markers were generally higher in the 20 μg/mL group, although the statistical significance of these differences varied among markers. Compared with the 20 μg/mL group, the expression levels of ScxA and Col1a1 were significantly higher in the 40 μg/mL group. Compared with the Control group, the mRNA expression levels of the osteogenic lineage-associated markers Osx and RUNX2, the chondrogenic lineage-associated marker SOX9, and the adipogenic lineage-associated marker PPARγ were significantly decreased in all three PRP-EV-treated groups. Compared with the 5 μg/mL group, the expression levels of Osx, RUNX2, and PPARγ were numerically lower in the 20 μg/mL group, but these differences were not statistically significant after Bonferroni correction. Compared with the 20 μg/mL group, the expression levels of Osx and PPARγ were numerically lower in the 40 μg/mL group, although these differences were not statistically significant after correction. In contrast, RUNX2 expression remained significantly lower in the 40 μg/mL group than in the 20 μg/mL group.

#### 3.8.2. Effects of PRP-EVs on the Protein Expression of Lineage-Associated Markers

WB was performed to further evaluate the effects of different concentrations of PRP-EVs on the protein expression levels of lineage-associated markers in TSCs.

**Figure 14:**
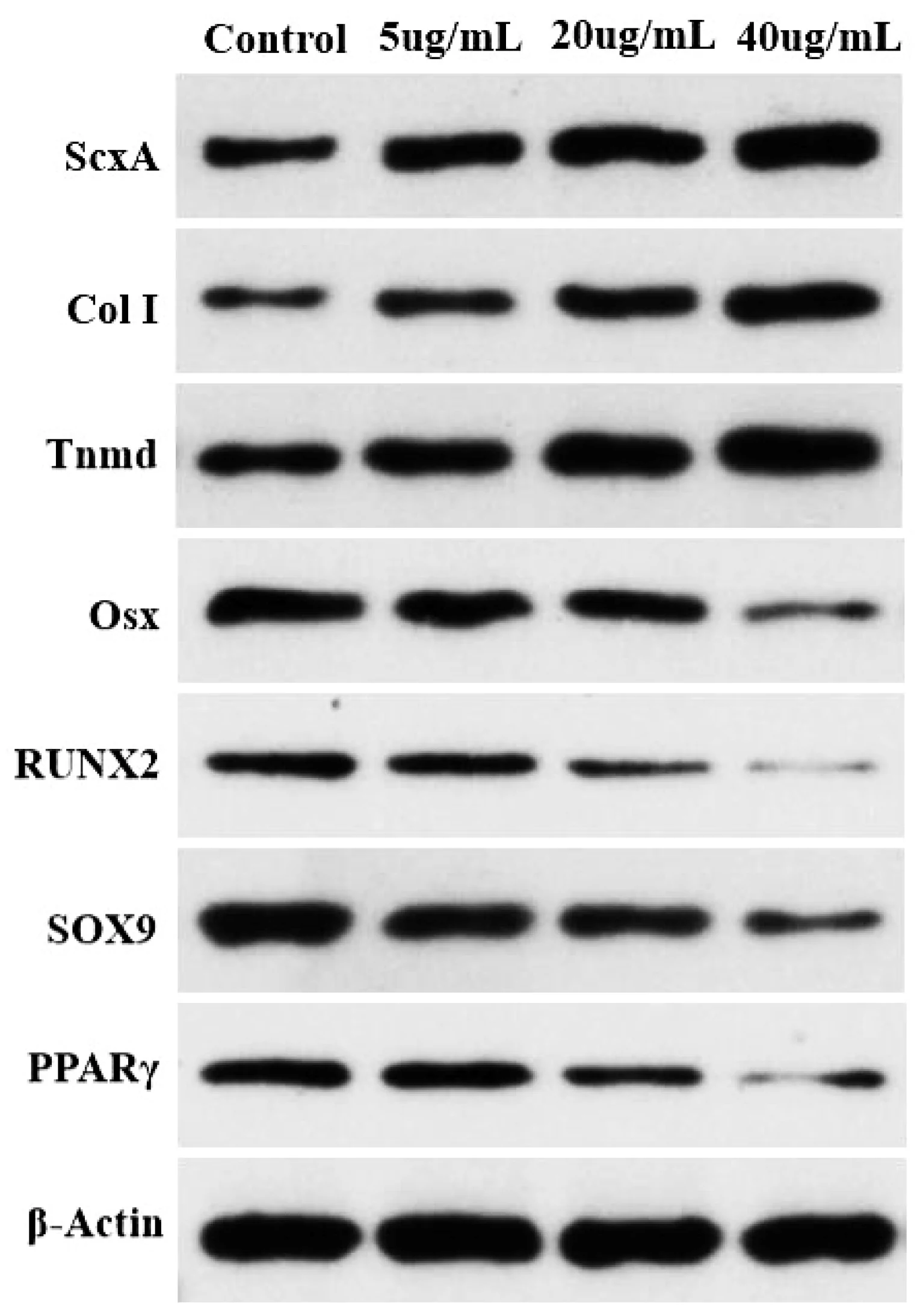
Representative Western blot images of lineage-associated markers in TSCs following treatment with different concentrations of PRP-EVs. The four columns represent the Control, 5, 20, and 40 μg/mL groups, respectively. Each group included three independently treated T25 flasks derived from a single pooled TSC preparation.

As shown in Figure 14 and Table 6 and Table 7, compared with the Control group, the protein expression levels of the tendon-associated markers ScxA, Col I, and Tnmd were generally higher in the PRP-EV-treated groups. For ScxA, significant increases were observed in the 20 and 40 μg/mL groups, whereas the increase in the 5 μg/mL group was not statistically significant. For Col I, significant increases were observed in the 20 and 40 μg/mL groups, while the difference between the 5 μg/mL and Control groups was not statistically significant after Bonferroni correction. Tnmd expression was significantly increased in the 20 and 40 μg/mL groups relative to the Control group. Compared with the 5 μg/mL group, the expression levels of all three markers were higher in the 20 and 40 μg/mL groups, with significant differences between the 40 μg/mL and 5 μg/mL groups. Although the expression levels of ScxA, Col I, and Tnmd were numerically higher in the 40 μg/mL group than in the 20 μg/mL group, none of these differences was statistically significant after Bonferroni correction.

Compared with the Control group, the protein expression levels of the osteogenic lineage-associated markers Osx and RUNX2 were lower in all three PRP-EV-treated groups, although statistically significant differences were observed only in comparisons of the 20 and 40 μg/mL groups with the Control group. Compared with the 5 μg/mL group, Osx expression was lower in both the 20 and 40 μg/mL groups; however, the difference between the 20 μg/mL and 5 μg/mL groups was not statistically significant after Bonferroni correction, whereas the difference between the 40 μg/mL and 5 μg/mL groups remained significant. RUNX2 expression was significantly lower in both the 20 and 40 μg/mL groups than in the 5 μg/mL group. Compared with the 20 μg/mL group, Osx expression was numerically lower in the 40 μg/mL group, but the difference was not statistically significant after correction. In contrast, RUNX2 expression remained significantly lower in the 40 μg/mL group than in the 20 μg/mL group.

Compared with the Control group, the protein expression levels of the chondrogenic lineage-associated marker SOX9 were significantly lower in all PRP-EV-treated groups. Compared with the 5 μg/mL group, SOX9 expression was lower in the 20 and 40 μg/mL groups, with a statistically significant difference between the 40 μg/mL and 5 μg/mL groups. SOX9 expression was also significantly lower in the 40 μg/mL group than in the 20 μg/mL group.

Compared with the Control group, the protein expression levels of the adipogenic lineage-associated marker PPARγ were lower in all three PRP-EV-treated groups, with statistically significant differences observed in comparisons of the 20 and 40 μg/mL groups with the Control group. Compared with the 5 μg/mL group, PPARγ expression was significantly lower in both the 20 and 40 μg/mL groups. PPARγ expression was further decreased in the 40 μg/mL group compared with the 20 μg/mL group.

Overall, the protein expression patterns of these lineage-associated markers were generally consistent with the corresponding mRNA expression patterns. Uncropped WB images are provided in Supplementary Figure S2.

## 4. Discussion

### 4.1. TSC Characterization and Experimental Model Validation

In the present study, cells isolated from rat Achilles tendons exhibited phenotypic and functional features commonly used to characterize TSCs. Flow cytometry showed that 89.4% and 100% of the analyzed cells were positive for CD44 and CD90, respectively, whereas immunofluorescence staining showed no detectable CD34 expression and positive staining for the tendon-associated markers ScxA, Tnmd, and Col I. These findings were consistent with previous characterization of rat TSCs, which identified a population expressing CD44 and CD90 but not CD34 and exhibiting tendon-associated properties [19]. A more recent study of rat Achilles TSCs similarly reported a mesenchymal stem cell-like phenotype, clonogenicity, multilineage differentiation potential, and tendon-related characteristics [20].

The identification of TSCs cannot be reliably based on a single surface or intracellular marker, as many stem cell-associated markers are also expressed by other mesenchymal cell populations. Previous studies have therefore emphasized the combined assessment of mesenchymal stromal cell-associated, stem cell-associated, and tendon-related markers, while recognizing the limitations of individual markers [24]. In the present study, the expression of both stem cell-associated and tendon-associated markers provided complementary phenotypic evidence for the characterization of the isolated cells. Their colony-forming capacity further supported their clonogenic and progenitor-like properties. Moreover, the observed osteogenic, chondrogenic, and adipogenic differentiation responses were consistent with the multilineage differentiation potential commonly described in TSCs [25].

The biological relevance of TSCs extends beyond their phenotypic characteristics, as these cells have been implicated in tendon homeostasis and repair. Previous research has associated TSCs with tenocyte proliferation, collagen production, and extracellular matrix remodeling during tendon healing, while dysregulated differentiation has been proposed to contribute to pathological tendon remodeling [10]. Establishing a tendon-derived cell population exhibiting both stem/progenitor-like characteristics and tendon-associated properties was therefore important for the subsequent intervention experiments. Collectively, the phenotypic and functional findings supported the use of this tendon-derived cell population as an in vitro model for investigating the cellular responses of TSCs to PRP-EVs.

### 4.2. PRP-EV Characterization

Characterization of the PRP-EV preparation provided a basis for interpreting the subsequent experiments. In the present study, TEM revealed vesicle-like structures, and DLS showed a mean particle diameter of 198.6 ± 94.5 nm. Western blotting detected the EV-associated proteins TSG101, CD9, and CD81, which are commonly used in the characterization of EV preparations [26]. These morphological, physical, and molecular findings provided complementary evidence supporting the enrichment of EVs in the PRP-derived preparation. Consistent with current recommendations, the characterization combined multiple analytical approaches rather than relying on a single feature to identify EVs [27].

Differential ultracentrifugation was used to isolate PRP-derived vesicles, as this approach is widely applied in EV research [28]. In combination with TEM, DLS, and immunoblotting, this method provided a basis for characterizing the preparation used in the subsequent TSC experiments. These findings supported the use of the preparation for investigating the cellular responses to PRP-EVs, while not establishing the identity or purity of every particle in the preparation.

### 4.3. PRP-EVs Increase the CCK-8 Signal in TSCs

The present study showed that PRP-EV treatment increased the CCK-8 signal in TSCs, although the magnitude of the response varied across concentrations and time points. The 20 and 40 μg/mL groups generally showed higher signals than the Control group, whereas the response at 5 μg/mL was less pronounced. The 40 μg/mL group generally exhibited the strongest response among the concentrations tested, although not all pairwise differences between concentrations were statistically significant.

These findings are consistent with previous studies investigating the effects of platelet-derived EVs on tendon progenitor cells. Previous work showed that PRP-EVs could be taken up by TSPCs and promote their proliferative activity [29]. More recently, PRP-EVs were reported to enhance TDSC proliferation under inflammatory conditions, accompanied by increased migration and tenogenic differentiation [18]. Platelet-derived EVs have also been reported to preserve the proliferative capacity of TSPCs under oxidative stress and senescence-related conditions, alongside improved tendon–bone regeneration in vivo [17]. Although these studies differed in cell models, experimental conditions, and EV preparations, their findings collectively support the potential of platelet-derived EVs to influence the biological activity of tendon progenitor cells.

The variation in CCK-8 signals across the tested concentrations is also noteworthy. The generally higher signals observed in the 20 and 40 μg/mL groups suggest that the response to PRP-EVs may vary with concentration. Similar concentration-related variation in EV-mediated effects has been reported in other cell systems, although the magnitude and direction of these effects can depend on the cell type and concentration range [30]. The stronger CCK-8 signals observed at higher concentrations may reflect greater exposure to biologically active components in the PRP-EV preparation. Among the concentrations evaluated, 40 μg/mL produced the highest overall CCK-8 signal, representing the strongest response observed under the experimental conditions tested.

### 4.4. PRP-EVs Increase Tendon-Associated Marker Expression in TSCs

Beyond the changes in CCK-8 signal, PRP-EV treatment was associated with increased expression of tendon-associated markers in TSCs. The mRNA expression levels of ScxA, Tnmd, and Col1a1 and the protein expression levels of ScxA, Tnmd, and Col I were generally higher following treatment, particularly at 20 and 40 μg/mL. However, the magnitude and statistical significance of these changes varied among markers and concentrations. Overall, the findings suggest that PRP-EVs modulate the expression of genes and proteins associated with the tendon phenotype of TSCs [31].

TSCs possess intrinsic tendon-associated characteristics, and previous research has shown that tendon-derived stem/progenitor cells can acquire or maintain tenogenic features during in vitro culture, accompanied by changes in the expression of tendon-related transcription factors and extracellular matrix components [32]. This property may contribute to their potential role in tendon maintenance and repair. In this context, the increased expression of tendon-associated markers observed in the present study may reflect modulation of endogenous tendon-related molecular programs following PRP-EV treatment.

The concurrent changes in ScxA, Tnmd, and Col I are biologically relevant because these markers represent different aspects of the tendon-associated phenotype. Scx is an important transcriptional regulator of tendon development and lineage specification, whereas Tnmd is associated with tendon cell characteristics, and type I collagen is a major component of tendon extracellular matrix [33]. Nevertheless, the expression of these markers is not exclusive to mature tenocytes and may vary according to cell source and culture conditions [21]. Their concurrent increases at both the mRNA and protein levels therefore provide complementary molecular evidence that PRP-EV treatment alters the tendon-associated phenotype of TSCs.

These findings are consistent with previous evidence that PRP-EVs can promote tenogenic differentiation-associated responses in TSPCs under inflammatory conditions and improve tendon repair in experimental tendinopathy [18]. TSC populations exhibit heterogeneity in their proliferative and multilineage differentiation capacities, which may influence their tendon-associated properties [34]. The changes observed in the present study may therefore represent a shift in the molecular profile of TSCs following PRP-EV exposure. One possible explanation is that EV-associated molecular cargo influences gene expression in recipient cells. Extracellular vesicles can transfer proteins, lipids, and nucleic acids to recipient cells and thereby modulate cellular functions. Transcriptomic evidence from previous research has also identified molecular changes associated with PRP-EV-mediated tenogenic responses in TDSCs [18]. These findings provide a plausible context for interpreting the marker-expression changes observed here, although the specific molecular events responsible for these changes remain to be elucidated.

### 4.5. Prp-Evs Reduce Alternative Lineage-Associated Marker Expression in TSCs

In addition to changes in tendon-associated markers, PRP-EV treatment was associated with reduced expression of markers related to alternative lineages in TSCs. At the mRNA level, the osteogenic markers Osx and RUNX2, the chondrogenic marker SOX9, and the adipogenic marker PPARγ were significantly downregulated in all PRP-EV-treated groups compared with the Control group. At the protein level, however, the significance of these changes varied among markers and concentrations. SOX9 expression was significantly reduced in all three treatment groups, whereas significant reductions in Osx, RUNX2, and PPARγ were observed primarily at 20 and 40 μg/mL. Moreover, some differences between treatment concentrations did not remain significant after Bonferroni correction. Overall, these findings indicate that PRP-EV treatment altered the expression of markers associated with osteogenic, chondrogenic, and adipogenic lineages, with the observed responses varying across markers and concentrations.

Although multilineage differentiation is an important characteristic of TSCs, their differentiation potential is heterogeneous. Clonal TSC populations have been shown to exhibit distinct combinations of proliferative and multilineage differentiation capacities, indicating substantial variation among individual TSC populations [34]. Under pathological conditions, this heterogeneity may be accompanied by changes in lineage-associated properties that are relevant to tendon remodeling. For example, TDSCs isolated from tendinopathic tendons have been reported to exhibit enhanced osteogenic and chondrogenic potential alongside reduced expression of tendon-associated markers, suggesting a relationship between altered TSC properties and impaired tendon healing [35]. Chondrogenic changes have also been observed in human Achilles tendinopathy and associated with pathological remodeling of the tendon matrix [36]. Furthermore, TDSCs from different experimental tendinopathy models have demonstrated distinct osteogenic and adipogenic potentials, supporting the view that the pathological microenvironment can influence the differentiation behavior of tendon-derived stem cells [37]. Studies targeting abnormal TDSC differentiation have likewise shown that modulating osteogenic or adipogenic processes can alter lineage-associated marker expression and, in some experimental settings, favor tendon-related characteristics [38]. Research on tendon heterotopic ossification has further implicated aberrant chondrogenic and osteogenic differentiation of TSPCs in ectopic tissue formation, with trauma and inflammatory signals potentially contributing to shifts away from normal tendon repair [39]. These findings highlight the relevance of lineage-associated changes to the biological behavior of TSCs and the development of tendon pathology.

In this context, the reduced expression of alternative lineage-associated markers observed after PRP-EV treatment suggests that PRP-EVs may influence the molecular profile of TSCs beyond tendon-associated pathways. The concurrent changes in osteogenic, chondrogenic, and adipogenic markers provide evidence that PRP-EV exposure is associated with broader alterations in lineage-associated gene and protein expression. This observation complements the increases in tendon-associated markers reported above and supports the interpretation that PRP-EVs modulate multiple aspects of the molecular phenotype of cultured TSCs.

### 4.6. Limitations and Future Directions

Several limitations should be acknowledged. First, this in vitro study used rat TSCs, which may not fully reflect the injured tendon microenvironment. TSCs from three rats and PRP-EVs from ten rats were each pooled into a single preparation, limiting the assessment of biological variability despite the use of three independently treated flasks per group.

Second, although TEM, DLS, and Western blotting supported the presence of EV-associated characteristics, particle concentration, negative EV markers, and potential non-vesicular contaminants were not assessed. Doses were normalized by total protein rather than particle number, and only three concentrations (5, 20, and 40 μg/mL) were tested. Thus, the optimal dose and safety profile remain undetermined. Future studies should improve EV characterization and evaluate a broader dose range with dedicated safety assessments.

Third, CCK-8 signals and lineage-associated markers were assessed without direct proliferation measurements or post-treatment functional differentiation assays. Therefore, these findings do not independently establish increased cell number, functional tenogenic differentiation, or suppression of alternative lineage differentiation. Future studies should incorporate direct proliferation assays, functional differentiation tests, and tendon-related endpoints such as collagen production and extracellular matrix organization.

Finally, primer amplification efficiency, Western blot densitometric linearity, and β-actin stability were not independently validated, and the underlying regulatory mechanisms remain unclear. Further studies should strengthen assay validation and investigate relevant signaling pathways and EV-associated molecular cargo, followed by in vivo validation of their effects on tendon repair.

## 5. Conclusions

PRP-EV treatment increased the CCK-8 signal and altered the expression of tendon-associated and alternative lineage-associated markers in cultured rat TSCs. Specifically, tendon-associated markers (ScxA, Tnmd, and Col I) were generally upregulated, whereas osteogenic (Osx and RUNX2), chondrogenic (SOX9), and adipogenic (PPARγ) markers were generally downregulated. Among the concentrations tested, 40 μg/mL produced the most pronounced overall response, although differences between concentrations were not consistently significant. These findings indicate that PRP-EVs modulate the molecular phenotype of TSCs in vitro; their effects on functional differentiation and tendon repair remain to be established.

## Figures and Tables

**Table 1:** Colony formation efficiency of TSCs.

Inoculated Cells (Cells/Dish)	Number of Colonies	Colony Formation Efficiency (%)
100	57.0 ± 1.7	57.0 ± 1.70
250	129.0 ± 3.1	51.6 ± 1.24

Note: Values are presented as mean ± SD from three replicate dishes per seeding density (*n* = 3). Colony formation efficiency was calculated as the number of colonies divided by the number of cells seeded × 100%.

**Table 2:** Comparison of platelet concentrations in whole blood and PRP from 10 rats.

Sample	Blood (×10^9^/L)	PRP (×10^9^/L)	PRP/Blood
1	823	3390	4.17
2	785	3423	4.36
3	952	3846	4.04
4	748	3373	4.51
5	765	3802	4.97
6	827	3705	4.48
7	997	4606	4.62
8	814	3492	4.29
9	776	3686	4.75
10	837	3633	4.34
Mean ± SD	832.40 ± 80.94	3695.60 ± 362.11	4.45 ± 0.28

Note: Platelet counts were measured separately in whole blood and PRP from each of the 10 rats before PRP-EV isolation. The 10 resulting PRP-EV preparations were subsequently pooled for characterization and experimental use.

**Table 3:** CCK-8 signal in TSCs following treatment with different concentrations of PRP-EVs.

Group	1 d	3 d	5 d	F	df_1_	df_2_	*p*	ηp^2^
Control	0.79 ± 0.02 ^▲◆^	0.78 ± 0.05 ^▲◆^	0.79 ± 0.01 ^▲◆^					
5 μg/mL	0.83 ± 0.03 ^▲^	0.80 ± 0.04 ^▲◆^	0.87 ± 0.06 ^▲^					
20 μg/mL	0.88 ± 0.03 ^■▲^	0.94 ± 0.02 ^▲^	0.98 ± 0.07 ^▲^					
40 μg/mL	0.95 ± 0.02 ^■^	1.08 ± 0.06 ^■^	1.25 ± 0.05					
Time main effect				43.741	2	16	<0.001	0.836
Group main effect				45.634	3	8	<0.001	0.945
Interaction effect				14.888	6	16	<0.001	0.848

Note: Data are presented as mean ± SD of blank-corrected OD450 values (*n* = 3 wells per group). The same wells were measured repeatedly on days 1, 3, and 5. All wells were seeded with TSCs from a single pooled preparation derived from three rats. For simple-effects comparisons of time, ^■^ indicates a significant difference from day 5 within the same treatment group. For between-group comparisons at each time point, ^▲^ indicates a significant difference from the 40 μg/mL group, and ^◆^ indicates a significant difference from the 20 μg/mL group. All pairwise comparisons were Bonferroni-adjusted, and differences were considered statistically significant at an adjusted *p* value of less than 0.05.

**Table 4:** Relative mRNA expression levels of lineage-associated markers in TSCs following treatment with different concentrations of PRP-EVs.

Marker	Group	Flask 1	Flask 2	Flask 3	Mean ± SD	F(3, 8)	*p*
ScxA	Control	1.00	1.22	1.45	1.22 ± 0.22	108.281	<0.001
	5 μg/mL	2.54	1.91	2.38	2.28 ± 0.33		
	20 μg/mL	5.44	4.86	6.35	5.44 ± 0.59 ^★☆^		
	40 μg/mL	9.40	7.91	8.06	8.46 ± 0.82 ^★☆▲^		
Col I	Control	1.00	1.25	1.52	1.26 ± 0.26	42.531	<0.001
	5 μg/mL	2.21	1.83	2.73	2.26 ± 0.49		
	20 μg/mL	4.05	4.80	3.56	4.13 ± 0.63 ^★☆^		
	40 μg/mL	4.97	6.25	5.68	5.43 ± 0.64 ^★☆▲^		
Tnmd	Control	1.00	0.85	1.11	0.99 ± 0.13	15.347	0.001
	5 μg/mL	2.54	2.12	2.02	2.23 ± 0.27		
	20 μg/mL	3.00	3.64	2.20	2.94 ± 0.72 ^★^		
	40 μg/mL	3.18	4.83	4.64	4.22 ± 0.90 ^★☆^		
Osx	Control	1.00	1.16	0.94	1.03 ± 0.11	26.427	<0.001
	5 μg/mL	0.74	0.60	0.86	0.73 ± 0.13 ^★^		
	20 μg/mL	0.51	0.63	0.51	0.55 ± 0.07 ^★^		
	40 μg/mL	0.34	0.36	0.42	0.35 ± 0.04 ^★☆^		
RUNX2	Control	1.00	1.07	1.13	1.07 ± 0.07	40.687	<0.001
	5 μg/mL	0.71	0.81	0.67	0.73 ± 0.07 ^★^		
	20 μg/mL	0.60	0.41	0.65	0.55 ± 0.13 ^★^		
	40 μg/mL	0.35	0.22	0.20	0.26 ± 0.08 ^★☆▲^		
SOX9	Control	1.00	0.88	1.00	0.96 ± 0.07	41.799	<0.001
	5 μg/mL	0.70	0.64	0.80	0.71 ± 0.08 ^★^		
	20 μg/mL	0.60	0.62	0.72	0.65 ± 0.06 ^★^		
	40 μg/mL	0.40	0.37	0.40	0.39 ± 0.02 ^★☆▲^		
PPARγ	Control	1.00	1.18	0.94	1.04 ± 0.12	36.907	<0.001
	5 μg/mL	0.87	0.72	0.74	0.78 ± 0.08 ^★^		
	20 μg/mL	0.55	0.61	0.65	0.60 ± 0.05 ^★^		
	40 μg/mL	0.36	0.39	0.40	0.38 ± 0.02 ^★☆^		

Note: Relative mRNA expression was calculated using the 2^−ΔΔ*CT*^ method, with the first sample in the Control group as the calibrator. Each cDNA sample was analyzed in technical triplicate, and the mean of the technical replicates was used for subsequent analysis. Values from three independently treated T25 flasks per group (*n* = 3 flasks per group) are presented individually and as mean ± SD. ^★^ indicates a significant difference compared with the Control group; ^☆^ indicates a significant difference compared with the 5 μg/mL group; ^▲^ indicates a significant difference compared with the 20 μg/mL group (Bonferroni-adjusted comparisons, *p* < 0.05).

**Table 5:** Pairwise comparisons of relative mRNA expression levels of lineage-associated markers among treatment groups.

Marker	Group Comparison (I − J)	Mean Difference (I − J)	SE	*p*	95% CI Lower	95% CI Upper
ScxA	Control − 5 μg/mL	−1.06	0.44	0.270	−2.604	0.492
	Control − 20 μg/mL	−4.22	0.44	<0.001	−5.770	−2.675
	Control − 40 μg/mL	−7.23	0.44	<0.001	−8.782	−5.687
	5 μg/mL − 20 μg/mL	−3.17	0.44	0.001	−4.714	−1.619
	5 μg/mL − 40 μg/mL	−6.18	0.44	<0.001	−7.726	−4.631
	20 μg/mL − 40 μg/mL	−3.01	0.44	0.001	−4.560	−1.465
Col I	Control − 5 μg/mL	−1.00	0.42	0.274	−2.470	0.471
	Control − 20 μg/mL	−2.88	0.42	0.001	−4.347	−1.406
	Control − 40 μg/mL	−4.38	0.04	<0.001	−5.846	−2.905
	5 μg/mL − 20 μg/mL	−1.88	0.42	0.013	−3.348	−0.407
	5 μg/mL − 40 μg/mL	−3.38	0.42	<0.001	−4.847	−1.906
	20 μg/mL − 40 μg/mL	−1.50	0.42	0.045	−2.970	−0.029
Tnmd	Control − 5 μg/mL	−1.24	0.49	0.207	−2.937	0.456
	Control − 20 μg/mL	−1.96	0.49	0.023	−3.653	−0.260
	Control − 40 μg/mL	−3.23	0.49	0.001	−4.927	−1.534
	5 μg/mL − 20 μg/mL	−0.72	0.49	1.000	−2.413	0.980
	5 μg/mL − 40 μg/mL	−1.99	0.49	0.021	−3.686	−0.293
	20 μg/mL − 40 μg/mL	−1.27	0.49	0.186	−2.970	0.423
Osx	Control − 5 μg/mL	0.30	0.08	0.03	0.027	0.567
	Control − 20 μg/mL	0.48	0.08	0.002	0.212	0.752
	Control − 40 μg/mL	0.66	0.08	<0.001	0.390	0.929
	5 μg/mL − 20 μg/mL	0.19	0.08	0.264	−0.085	0.455
	5 μg/mL − 40 μg/mL	0.36	0.08	0.010	0.093	0.632
	20 μg/mL − 40 μg/mL	0.18	0.08	0.308	−0.092	0.447
RUNX2	Control − 5 μg/mL	0.33	0.08	0.013	0.073	0.595
	Control − 20 μg/mL	0.51	0.08	0.001	0.253	0.775
	Control − 40 μg/mL	0.81	0.08	<0.001	0.548	1.070
	5 μg/mL − 20 μg/mL	0.18	0.08	0.26	−0.081	0.441
	5 μg/mL − 40 μg/mL	0.47	0.08	0.001	0.214	0.736
	20 μg/mL − 40 μg/mL	0.30	0.08	0.026	0.034	0.556
SOX9	Control − 5 μg/mL	0.25	0.05	0.008	0.069	0.427
	Control − 20 μg/mL	0.31	0.05	0.002	0.136	0.493
	Control − 40 μg/mL	0.57	0.05	<0.001	0.393	0.751
	5 μg/mL − 20 μg/mL	0.07	0.05	1.000	−0.113	0.245
	5 μg/mL − 40 μg/mL	0.32	0.05	0.001	0.145	0.502
	20 μg/mL − 40 μg/mL	0.26	0.05	0.006	0.079	0.436
PPARγ	Control − 5 μg/mL	0.26	0.06	0.022	0.037	0.487
	Control − 20 μg/mL	0.44	0.06	0.001	0.216	0.666
	Control − 40 μg/mL	0.66	0.06	<0.001	0.431	0.880
	5 μg/mL − 20 μg/mL	0.18	0.06	0.145	−0.046	0.404
	5 μg/mL − 40 μg/mL	0.39	0.06	0.002	0.169	0.619
	20 μg/mL − 40 μg/mL	0.21	0.06	0.064	−0.011	0.439

Note: Pairwise comparisons were performed among the Control, 5, 20, and 40 μg/mL PRP-EV groups following one-way ANOVA. Mean differences are calculated as the mean of group I minus the mean of group J, as indicated in the Group comparison column. SE denotes standard error. *p* values are Bonferroni-adjusted for multiple pairwise comparisons within each marker. The lower and upper limits represent the corresponding 95% confidence interval for the mean difference. Each group comprised three independently treated T25 flasks, all derived from a single pooled TSC preparation from three rats.

**Table 6:** Relative protein expression levels of lineage-associated markers in TSCs following treatment with different concentrations of PRP-EVs.

Marker	Group	Flask 1	Flask 2	Flask 3	Mean ± SD	F(3, 8)	*p*
ScxA	Control	0.28	0.39	0.46	0.38 ± 0.09	35.075	<0.001
	5 μg/mL	0.53	0.70	0.68	0.64 ± 0.10 ^★^		
	20 μg/mL	0.70	0.83	0.77	0.77 ± 0.07 ^★^		
	40 μg/mL	0.98	1.08	1.12	1.06 ± 0.07 ^★☆^		
Col I	Control	0.26	0.26	0.46	0.33 ± 0.11	12.785	0.002
	5 μg/mL	0.38	0.58	0.62	0.53 ± 0.13		
	20 μg/mL	0.54	0.74	0.71	0.66 ± 0.11 ^★^		
	40 μg/mL	0.80	0.93	0.82	0.85 ± 0.07 ^★☆^		
Tnmd	Control	0.36	0.48	0.59	0.48 ± 0.12	13.631	0.002
	5 μg/mL	0.52	0.75	0.72	0.66 ± 0.13		
	20 μg/mL	0.71	0.94	0.80	0.82 ± 0.12 ^★^		
	40 μg/mL	1.00	1.13	0.99	1.04 ± 0.08 ^★☆^		
Osx	Control	0.68	0.77	0.85	0.77 ± 0.08	31.633	<0.001
	5 μg/mL	0.54	0.71	0.74	0.67 ± 0.11		
	20 μg/mL	0.49	0.45	0.54	0.49 ± 0.05 ^★^		
	40 μg/mL	0.21	0.26	0.20	0.22 ± 0.03 ^★☆▲^		
RUNX2	Control	0.51	0.47	0.48	0.49 ± 0.02	62.335	<0.001
	5 μg/mL	0.51	0.47	0.39	0.46 ± 0.06		
	20 μg/mL	0.30	0.23	0.23	0.25 ± 0.04 ^★☆^		
	40 μg/mL	0.11	0.12	0.10	0.11 ± 0.01 ^★☆▲^		
SOX9	Control	0.93	0.85	0.85	0.88 ± 0.04	46.547	<0.001
	5 μg/mL	0.53	0.68	0.62	0.61 ± 0.08 ^★^		
	20 μg/mL	0.48	0.62	0.49	0.53 ± 0.08 ^★^		
	40 μg/mL	0.25	0.31	0.23	0.27 ± 0.04 ^★☆▲^		
PPARγ	Control	0.95	0.92	0.99	0.95 ± 0.03	27.556	<0.001
	5 μg/mL	0.82	0.92	0.89	0.88 ± 0.05		
	20 μg/mL	0.52	0.67	0.54	0.58 ± 0.08 ^★☆^		
	40 μg/mL	0.47	0.62	0.43	0.51 ± 0.10 ^★☆^		

Note: Protein band intensities were quantified using ImageJ 1.54g version and normalized to β-actin. Values from three independently treated T25 flasks per group (*n* = 3 flasks per group) are presented individually and as mean ± SD. All flasks were seeded with TSCs from a single pooled preparation derived from three rats. ^★^ indicates a significant difference compared with the Control group; ^☆^ indicates a significant difference compared with the 5 μg/mL group; ^▲^ indicates a significant difference compared with the 20 μg/mL group (Bonferroni-adjusted comparisons, *p* < 0.05).

**Table 7:** Pairwise comparisons of relative protein expression levels of lineage-associated markers among treatment groups.

Marker	Group Comparison (I − J)	Mean Difference (I − J)	SE	*p*	95% CI Lower	95% CI Upper
ScxA	Control − 5 μg/mL	−0.26	0.07	0.029	−0.497	−0.026
	Control − 20 μg/mL	−0.39	0.07	0.002	−0.629	−0.158
	Control − 40 μg/mL	−0.68	0.07	<0.001	−0.917	−0.446
	5 μg/mL − 20 μg/mL	−0.13	0.07	0.515	−0.368	0.103
	5 μg/mL − 40 μg/mL	−0.42	0.07	0.002	−0.656	−0.185
	20 μg/mL − 40 μg/mL	−0.29	0.07	0.17	−0.523	−0.523
Col I	Control − 5 μg/mL	−0.20	0.09	0.303	−0.503	0.103
	Control − 20 μg/mL	−0.34	0.09	0.028	−0.640	−0.035
	Control − 40 μg/mL	−0.52	0.09	0.002	−0.824	−0.218
	5 μg/mL − 20 μg/mL	−0.14	0.09	0.922	−0.440	0.166
	5 μg/mL − 40 μg/mL	−0.32	0.09	0.037	−0.624	−0.018
	20 μg/mL − 40 μg/mL	−0.18	0.09	0.406	−0.487	0.119
Tnmd	Control − 5 μg/mL	−0.19	0.09	0.456	−0.502	0.131
	Control − 20 μg/mL	−0.34	0.09	0.035	−0.655	−0.022
	Control − 40 μg/mL	−0.56	0.09	0.002	−0.878	−0.244
	5 μg/mL − 20 μg/mL	−0.15	0.09	0.791	−0.469	0.164
	5 μg/mL − 40 μg/mL	−0.38	0.09	0.020	−0.692	−0.059
	20 μg/mL − 40 μg/mL	−0.22	0.09	0.240	−0.540	0.094
Osx	Control − 5 μg/mL	0.10	0.06	0.788	−0.107	0.308
	Control − 20 μg/mL	0.27	0.06	0.011	0.064	0.479
	Control − 40 μg/mL	0.54	0.06	<0.001	0.335	0.751
	5 μg/mL − 20 μg/mL	0.17	0.06	0.126	−0.037	0.379
	5 μg/mL − 40 μg/mL	0.44	0.06	<0.001	0.235	0.650
	20 μg/mL − 40 μg/mL	0.27	0.06	0.011	0.064	0.479
RUNX2	Control − 5 μg/mL	0.03	0.03	1.000	−0.083	0.139
	Control − 20 μg/mL	0.23	0.03	<0.001	0.123	0.344
	Control − 40 μg/mL	0.37	0.03	<0.001	0.264	0.485
	5 μg/mL − 20 μg/mL	0.21	0.03	0.001	0.095	0.316
	5 μg/mL − 40 μg/mL	0.35	0.03	<0.001	0.236	0.457
	20 μg/mL − 40 μg/mL	0.14	0.03	0.013	0.031	0.252
SOX9	Control − 5 μg/mL	0.27	0.05	0.006	0.084	0.447
	Control − 20 μg/mL	0.35	0.05	0.001	0.165	0.528
	Control − 40 μg/mL	0.61	0.05	<0.001	0.430	0.793
	5 μg/mL − 20 μg/mL	0.08	0.05	0.974	−0.101	0.262
	5 μg/mL − 40 μg/mL	0.35	0.05	0.001	0.164	0.527
	20 μg/mL − 40 μg/mL	0.26	0.05	0.06	0.084	0.447
PPARγ	Control − 5 μg/mL	0.08	0.06	1.000	−0.129	0.281
	Control − 20 μg/mL	0.37	0.06	0.001	0.170	0.580
	Control − 40 μg/mL	0.44	0.06	<0.001	0.240	0.650
	5 μg/mL − 20 μg/mL	0.30	0.06	0.006	0.094	0.504
	5 μg/mL − 40 μg/mL	0.37	0.06	0.001	0.164	0.574
	20 μg/mL − 40 μg/mL	0.07	0.06	1.000	−0.135	0.275

Note: Pairwise comparisons were performed among the Control, 5, 20, and 40 μg/mL PRP-EV groups following one-way ANOVA. Mean differences are calculated as the mean of group I minus the mean of group J, as indicated in the Group comparison column. SE denotes standard error. *p* values are Bonferroni-adjusted for multiple pairwise comparisons within each marker. The lower and upper limits represent the corresponding 95% confidence interval for the mean difference. Each group comprised three independently treated T25 flasks, all derived from a single pooled TSC preparation from three rats.

## Data Availability

The original data supporting the findings of this study are included in the Supplementary Materials. Further inquiries can be directed to the corresponding author.

## Supplementary Materials

The following supporting information can be downloaded at: https://www.mdpi.com/article/10.3390/cells15212008/s1.
