# Supplementary material for: Effects of Different Concentrations of Platelet-Rich Plasma-Derived Extracellular Vesicles on Cellular Activity and Lineage-Associated Marker Expression in Tendon Stem Cells
Source: Cells. 2026 Nov 4;15(21):2008. doi: 10.3390/cells15212008 (PMC13761721; doi:10.3390/cells15212008)
Supplement: Supplementary file 1 [file cells-15-02008-s001.zip › Supplementary Table.pdf]

Supplementary Table S1. Antibody

Antibody target	Expected molecular weight (kDa)	Manufacturer	Country	Catalog No.	Working dilution	Manufacturer's datasheet
TSG101 primary antibody	41	Abcam	UK	AB125011	1: 1000	https://www.abcam.cn/products/primary-antibodies/tsg101-antibody-epr7130b-ab125011
CD9 primary antibody	22	Abcam	UK	AB236630	1: 1000	https://www.abcam.cn/products/primary-antibodies/cd9-antibody-epr23105-121-ab236630
CD81 primary antibody	24	Abcam	UK	AB79559	1: 1000	https://www.abcam.cn/products/primary-antibodies/cd81-antibody-m38-ab79559#1
ScxA primary antibody	22	Abcam	UK	AB58655	1: 1200	https://www.abcam.cn/products/primary-antibodies/scxa-antibody-ab58655
Col I primary antibody	139	Beijing Bioss	China	BS10423R	1: 1000	https://www.bioss.com.cn/productDetail?goods_id=8236
Tnmd primary antibody	37	Beijing Bioss	China	BS7525R	1: 1000	https://www.bioss.com.cn/productDetail?goods_id=27536
Osx primary antibody	45	Affinity	USA	DF7731	1: 1000	https://affbiotech.cn/goods-11466-DF7731-osterix_Antibody.html
RUNX2 primary antibody	57	Affinity	USA	AF5186	1: 1000	https://affbiotech.cn/goods-4493-AF5186-RUNX2_Antibody.html
SOX9 primary antibody	56	Affinity	USA	AF6330	1: 1000	https://affbiotech.cn/goods-1934-AF6330-SOX9_Antibody.html
PPARγ primary antibody	58	Affinity	USA	AF6284	1: 1000	https://affbiotech.cn/goods-1883-AF6284-PPAR_gamma_Antibody.html
β-actin primary antibody	42	Affinity	USA	AF7018	1: 1600	https://affbiotech.cn/goods-6288-AF7018-beta_Actin_Antibody.html
Goat anti-rabbit secondary antibody	N/A	ZSGB-BIO	China	ZB2301	1: 10,000	http://www.zsbio.com/product/ZB-2301
Goat anti-mouse secondary antibody	N/A	ZSGB-BIO	China	ZB2305	1: 10,000	http://www.zsbio.com/product/ZB-2305

Supplementary Table S2. Primer sequences and amplicon lengths used for qPCR

Gene	Genbank accession	Gene sequence(5'-3')	bp
Col 1a1	NM_053304	Sense: TGGCAACCTCAAGAAGTCCC	99
		Anti-sense:CCATCCACAAGCGTGCTGTA	
Tnmd	NM_022290	Sense:CAGGACTTTGAGGAGGATGG	132
		Anti-sense:CTCGCTTGCTTGTCTGGTGC	
ScxA	NM_001130508	Sense:TGGGCAACGTGCTACTGGTG	165
		Anti-sense:GTTTGGGCTGGGTGTTCTCG	
Osx	NM_001037632	Sense:GCCTACTTACCCGTCTGACTTTG	115
		Anti-sense:TGCCTTGGGCTTATAGACATCTT	
RUNX2	NM_001278483	Sense:GGACGAGGCAAGAGTTTCA	249
		Anti-sense:TGGTGCTGAGTTCAGGGAG	
PPARγ	NM_001145366	Sense:CCTTTACCACGGTTGATTTCTC	139
		Anti-sense:GGCTCTACTTTGATCGCACTTT	
SOX9	NM_080403.2	Sense:AACAGACGTGCAAGCTGGGAAAG	173

β-actin	NM_031144.3	Anti-sense:GCGGCAGGTATTGGTCAAACTCA<br>Sense:AGATTACTGCCCTGGCTCCTAG<br>Anti-sense:CATCGTACTCCTGCTTGCTGAT	144

Note: All primer sequences are presented in the 5′–3′ direction. GenBank accession numbers refer to the corresponding reference mRNA sequences, and amplicon lengths are reported in base pairs (bp).

Supplementary Table S3. Main experimental reagents

Reagent	Manufacturer	Country of origin	Catalog No.	Working dilution
Chloral hydrate	Shanghai Macklin Biochemical Technology Co., Ltd.	China	C804539	
PBS buffer	Procell	China	PB80327	
Type I collagenase	Solarbio	China	C8140	
Neutral protease	Solarbio	China	D6430	
α-MEM	Meilunbio	China	PWL007	
FBS	Gibco	USA	—	
1%P/S	Gibco	USA	—	
FITC anti-rat CD44 H (Pgp-1, H-CAM) Antibody	Biolegend	USA	203906	
PE anti-rat CD90/mouse CD90.1 Antibody	Biolegend	USA	205903	
0.25% Trypsin-EDTA	Meilunbio	China	MA0232	
TRITON X-100	Sigma	USA	T8787	
5% BSA blocking solution	Beijing Solarbio	China	SW3015	
DAPI staining solution	Boster	USA	AR1177	
Antifade mounting medium	Beijing ZSGB-BIO	China	ZLI-9556	
CD34 fluorescent primary antibody	Affinity	USA	AF5149	1: 120
NS fluorescent primary antibody	Beijing Bioss	China	bs-6846R	1: 150
SSEA-4 fluorescent primary antibody	Abcam	UK	ab16287	1: 150
Oct-3 fluorescent primary antibody	Beijing Bioss	China	bs-1111R	1: 100
ScxA fluorescent primary antibody	Beijing Bioss	China	bs-11364R	1: 120
Tnmd fluorescent primary antibody	Beijing Bioss	China	bs-7525R	1: 120
Col I fluorescent primary antibody	Beijing Bioss	China	bs-10423R	1: 100
Fluorescent secondary antibody	Beijing ZSGB-BIO	China	ZF0516	
0.1% Crystal violet staining solution	Solarbio	China	G1063	
4% Paraformaldehyde fixative	Biosharp	China	BL539SA	
1% Gelatin solution	Solarbio	China	G0040	
Rat tendon stem cell osteogenic differentiation induction kit	Suzhou Haixing Biotechnology	China	TSGU-D101	
Rat tendon stem cell chondrogenic differentiation induction kit	Suzhou Haixing Biotechnology	China	TSGU-D203	
Rat tendon stem cell adipogenic differentiation induction kit	Suzhou Haixing Biotechnology	China	TSGU-D102	
0.45 μm membrane filter	Millipore	USA	R6BA09493	

2% Phosphotungstic acid (freshly prepared)	—	—	—
BCA protein assay kit	Servicebio	China	G2026
RIPA lysis buffer	Solarbio	China	R0020
Protease inhibitor	Biosharp	China	BL507A
10% Precast gel	Shanghai Epizyme Biotechnology	China	LK303
5 × Protein loading buffer	Shanghai Epizyme Biotechnology	China	LT103
100% Methanol	Tianjin Yongda Chemical Reagent Co., Ltd.	China	—
Transfer buffer	Shanghai Epizyme Biotechnology	China	PS109
PVDF membrane	Millipore	USA	IPVH00010
1 × Protein-free rapid blocking solution	Shanghai Epizyme Biotechnology	China	PS108P
CCK-8 assay kit	Solarbio	China	CA1210
TRIzol reagent	Invitrogen	USA	15596-026
Chloroform	Tianjin Yongda Chemical Reagent Co., Ltd.	China	—
Isopropanol	Tianjin Yongda Chemical Reagent Co., Ltd.	China	—
Absolute ethanol	Tianjin Yongda Chemical Reagent Co., Ltd.	China	—
DEPC-treated water	Sigma	USA	31000-50ML
RNase-free ddH_2O	Solarbio	China	R1600
Tris-EDTA buffer	Baiolaibo	China	GL2294
HiFiScript Fast Genomic DNA Removal and cDNA First-Strand Synthesis Kit	CWBIO	China	CW2582M
Fluorescent quantitative PCR kit	Monad Biotech	China	MQ00401S
1 × TBST	Shanghai Epizyme Biotechnology	China	PS103S
X-ray film	Carestream	Canada	XBT
ECL detection reagent	ZSGB-BIO	China	sc-2048
Universal developer powder and acidic fixer powder	Tianjin Tianluhai Photosensitive Materials Co., Ltd.	China	—

Supplementary Table S4. Main experimental instruments

Instrument	Model	Manufacturer	Country of origin
Cell culture incubator	HF90	Heal Force	China
Laminar flow clean bench	GVD650	Shanghai SHULI	China
Flow cytometer	FC500	Beckman	USA
Inverted white-light microscope	DSZ2000X	UOP	China
Low-speed centrifuge	DM0412S	DLAB	China
Biological safety cabinet	SW-TFG-15	Guangzhou Ruizhi Purification Equipment	China
Drying oven	MIR-153	SANYO	Japan
Low-speed automatic balancing centrifuge	LDZ5-2	Beijing Jingli	China
Microtome	RM2135	Leica	Germany
Micropipette	Research plus	Eppendorf	Germany
Electronic analytical balance	AE100	METTER	Switzerland
Constant-temperature water bath	DSHZ-300	Jiangsu Taicang	China
Induction cooker	JYC-21ES55C	Joyoung	China

Fluorescence microscope	BX43	OLYMPUS	Japan
Imaging system	UC90	OLYMPUS	Japan
Paraffin microtome	RM2135	Leica	Germany
Cell counting chamber	177-112C	Waston	Japan
Benchtop refrigerated centrifuge	Microfuge 20R	Beckman	USA
Ultra-low-temperature freezer	905	Thermo	USA
Ultracentrifuge	CP100MX	Hitachi	Japan
Transmission electron microscope	HT7800/HT7700	Hitachi	Japan
150-mesh carbon-coated copper grid	D11023	Beijing Daji Keyi Technology Co., Ltd	China
Nanoparticle size and zeta potential analyzer	Zetasizer Nano-ZS 90	Malvern	UK
Rocking shaker	TSY-B	Servicebio	China
Microplate reader	ELx800	Biotek	USA
High-speed refrigerated centrifuge	Centrifuge 5417R	Eppendorf	Germany
Microvolume UV–Vis spectrophotometer	DS-11	Denovix	USA
Ultra-low-temperature freezer	MDF-U72V	SANYO	Japan
Ultrapure water purification system	—	Beijing Kangming Taike Technology	China
Electrophoresis apparatus	DYCZ-24DN	Beijing Liuyi Biotechnology	China
Rocking shaker	TS-8	Haimen Qilinbell Instrument Manufacturing	China
Scanner	V19	EPSON	Japan
Ultrasonic cell disruptor	TL-650Y	Jiangsu Tianling Instrument	China
Vortex mixer	QL-902	Haimen Qilinbell Instrument Manufacturing	China
Benchtop high-speed refrigerated centrifuge	Centrifuge 5415D	Eppendorf	Germany
Real-time PCR system	CFX Connect™	BIO-RAD	USA

Supplementary Table S5. qPCR reaction mixture

Component	Amount per reaction
2 × MonAmp™ ChemoHS qPCR Mix	10 μL
10 μM forward primer	0.6 μL
10 μM reverse primer	0.6 μL
cDNA	100 ng (≤ 2 μL)
RNase-free ddH_2O	To a final volume of 20 μL
